# Vaping and smoking trajectories among youth and adults in the United States across the period 2014–2022

**DOI:** 10.3389/fpubh.2026.1775015

**Published:** 2026-02-23

**Authors:** Karin A. Kasza, Maciej L. Goniewicz, Pete Driezen, David Hammond, Andrew J. Hyland

**Affiliations:** 1 Department of Health Behavior, Roswell Park Comprehensive Cancer Center, Buffalo, NY, United States; 2 School of Public Health Sciences, University of Waterloo, Waterloo, ON, Canada

**Keywords:** adults, cigarettes, e-cigarettes, longitudinal, population, trajectories, vaping, youth

## Abstract

**Introduction:**

Multiple occurrences in the United States may have impacted trajectories (i.e., transitions over time) in the use of electronic nicotine delivery systems (ENDS) and cigarette smoking in the population over time. We examine youths' trajectories in ENDS use and adults' trajectories in cigarette smoking across two periods: 2014–2016–2017; 2019–2021–2022.

**Methods:**

We analyzed longitudinal Population Assessment of Tobacco and Health (PATH) Study data from youth ages 12–17 in 2014 (*N* = 9,582), youth ages 12–17 in 2019 (*N* = 7,325), adults who smoked cigarettes in 2014 (*N* = 8,999), and adults who smoked cigarettes in 2019 (*N* = 5,800). We evaluated trajectories in ENDS use among youth and trajectories in cigarette smoking among adults, each across two periods: 2014–2016–2017; 2019–2021–2022.

**Results:**

Among youth, rates of progression from less frequent to frequent ENDS use were 23 and 35% across two periods: 2014–2016–2017; 2019–2021–2022, respectively; rates of maintaining frequent ENDS use were 13 and 51% across two periods: 2014–2016–2017; 2019–2021–2022, respectively. Among adults who smoked cigarettes and used ENDS frequently, 23 and 42% discontinued smoking across two periods: 2014–2016–2017; 2019–2021–2022, respectively.

**Conclusions:**

Across the period 2019–2021–2022, the number of youths in 2019 who used ENDS frequently in 2022 was 1.6 million, while the number of adults who smoked cigarettes in 2019 who used ENDS frequently in 2019, and stopped smoking cigarettes in 2022, was 1.6 million. The population-level impact of ENDS in the US appears greater since the significant evolution in the ENDS market, including the rise in popularity of salt-based nicotine ENDS, the coronavirus disease 2019 (COVID-19) pandemic, and various tobacco control policies.

## 1 Introduction

In the United States (US), the Center for Tobacco Products (CTPs) has the authority to authorize the marketing of tobacco products under the “Appropriate for the Protection of Public Health” (APPH) standard (1). This standard requires that regulators weigh the potential benefits against the potential harms of a product when making marketing authorization decisions. Electronic nicotine delivery systems (ENDS, also known as e-cigarettes) may pose both benefits and harms to the population, and the harm–benefit ratio may change over time as the products evolve. A recent population study in the US showed that after 2017, there was a greater increase in adults' cigarette discontinuation rates among those who used ENDS than among those who did not use ENDS. This correlative relationship was not present before 2016 (2). Additionally, cross-sectional studies since 2017 have shown an increasing trend in the prevalence of higher frequency ENDS use and nicotine dependence symptoms among youth who use ENDS (3).

Several occurrences in the US may have impacted ENDS use behaviors and transitions over time in cigarette smoking, and ENDS use behaviors at the population level. The market for ENDS expanded in the US in the past decade (4, 5). In 2016, salt-based nicotine e-liquid was introduced to the market by JUUL Labs, Inc., San Francisco, CA and grew in popularity in the years that followed (4, 6). By 2018, JUUL^®^ ENDS accounted for the majority of ENDS sales in the US (7). The salt-based nicotine formulation enabled more efficient delivery of higher nicotine concentrations to consumers than previous formulations that used free-base nicotine; salt-based nicotine causes a chemical change in the aerosol, which minimizes irritation and facilitates inhalation, particularly for aerosols with higher nicotine concentrations (8, 9). Salt-based nicotine ENDS product advancement may facilitate increased ENDS use among people who use ENDS, including youth and adults who smoke cigarettes, which may support transitions away from cigarette smoking.

The coronavirus disease 2019 (COVID-19) pandemic overlapped the period during which salt-based nicotine ENDS became popular in the US. National data show that there were decreases in the prevalence of tobacco use among youth at the beginning of the pandemic, which may have been due in part to peer separation (10). Findings on the impact of the COVID-19 pandemic on adults' cigarette smoking behaviors have been mixed (10, 11). Additionally, the period during which salt-based nicotine ENDS became popular was also a period of increased ENDS regulatory actions, including federal- (12) and state-level (13) ENDS flavor restrictions intended to reduce the appeal of ENDS among youth, as well as Tobacco 21 (14), designed to reduce youths' access to ENDS and tobacco products. Changes in ENDS industry marketing practices, such as advertising products without cartoons and disengaging from social media promotions (15), may also have affected ENDS use in the population. Finally, the salt nicotine ENDS period occurred after an outbreak of lung injuries initially attributed to ENDS use (EVALI) (16), which may have increased ENDS harm perceptions and decreased ENDS use among youth and adults (17).

Given multiple occurrences that happened after 2017 that may have affected vaping among youth and smoking behaviors among adults who do or do not vape, the purpose of this study was to describe trajectories in product use behaviors across two periods: 2014–2016–2017; 2019–2021–2022. We used nationally representative longitudinal data from the Population Assessment of Tobacco and Health (PATH) Study to examine trajectories of ENDS use among youth, including ENDS use initiation rates, rates of progressing to frequent use, and rates of maintaining ENDS use. Consideration of both youth and adults is needed to inform regulatory decisions that are based on an APPH standard (1). Thus, we also examine trajectories in cigarette smoking among adults while considering their ENDS use.

## 2 Materials and methods

### 2.1 Participants

We analyzed data from two PATH Study data collection periods, each spanning approximately 3 years. Among the periods, the first period spanned 2014–2016–2017 and consisted of participants interviewed in-person using audio computer-assisted self-interviews (ACASI) in the duration between September 2013 to December 2014 (Wave 1, “2014”), who were reinterviewed in person approximately 2 years later in the duration between October 2015 and October 2016 (Wave 3, “2016”), and then were reinterviewed in person again approximately 1 year later in the duration between December 2016 and January 2018 (Wave 4, “2017”) (18). The first period comprised youth ages 12–17 in 2014 (*N* = 9,582) and adults ages 18+ who smoked cigarettes in 2014 (*N* = 8,999). The Wave 2 data were excluded from the first set of data to improve timeframe comparability with the second set of data.

The second set of data spanned between 2019–2021–2022 and consisted of participants interviewed in-person using ACASI in the duration between December 2018 and November 2019 (Wave 5, “2019”), who were reinterviewed in-person using ACASI or by telephone using computer-assisted telephone interview (CATI) approximately 2 years later in the duration between March 2021 and November 2021 (Wave 6, “2021”), and then were reinterviewed again using in-person ACASI, telephone CATI, or web-based survey approximately 1 year later in the duration between January 2022 and April 2023 (Wave 7, “2022”) (18). The period 2019–2021–2022 comprised youth ages 12–17 in 2019 (*N* = 7,459) and adults ages 18+ who smoked cigarettes in 2019 (*N* = 5,800). Some behaviors are known to be underreported via telephone data collection compared to in-person data collection due to social desirability bias (19), and thus the 2021 results should be interpreted accordingly. For each period, individuals needed to participate in all three waves to be eligible for analyses. Individuals who met the eligibility criteria for both periods (e.g., adults who smoked cigarettes in 2014 and in 2019) contributed data for both periods. See Supplementary Table S1 for a description of the youth population, and see Supplementary Table S2 for a description of the adult population.

The PATH Study is conducted by Westat, Rockville, MD and approved by the Westat Institutional Review Board; the study reported here was approved by the Roswell Park Institutional Review Board. All youth participants ages 12–17 years provided assent, and a parent/legal guardian provided informed consent. Youth participants who became 18+ at follow-up could participate as adults. All adult participants ages 18+ years provided informed consent. The PATH Study employs a stratified, address-based, area-probability sampling design, and the weighted estimates reported here represent the US civilian non-institutionalized population. The weighted response rates ranged from 56% to 80% (18). Non-response bias analysis reports have been published for each wave of the PATH Study; while there were differences between those who did and did not respond in all waves (including differences by ethnicity and socioeconomic status), the weighting adjustments have been found to leave little potential for non-response to bias estimates. Extensive details on the PATH Study design, methods (20–22), and demographic and tobacco-use distributions (23) are published elsewhere. Details on interviewing procedures, questionnaires, sampling, weighting, response/attrition rates, non-response bias analyses, and access to PATH Study Public Use Files and PATH Study Restricted Use Files are available at https://doi.org/10.3886/Series606 (18).

The study reported in this manuscript used PATH Study Public Use data (24) from Waves 1–4 and PATH Study Restricted Use data (25) from Waves 5–7, with the latter being conducted following a Data Use Agreement between Roswell Park Comprehensive Cancer Center and the Inter-university Consortium for Political and Social Research National Addiction and HIV Data Archive Program.

### 2.2 Measures

#### 2.2.1 Youth

Among youth, we evaluated trajectories of ENDS use while accounting for the frequency of ENDS use. Participants in the PATH Study were asked: “Have you ever used an electronic nicotine product, even one or two times?” At each interview, participants were asked: “In the past 30 days, have you used an electronic nicotine product, even one or two times?” and among those who had used an electronic nicotine product, “On how many of the past 30 days did you use an electronic nicotine product?” For each interview wave, we created a four-level ENDS use variable as follows (regardless of other product use): (1) never use, (2) former use (responded yes to ever use and responded no to use in the past 30 days), (3) current less frequent use (used on 1–19 days in the past 30 days), and (4) current frequent use (used on 20+ days in the past 30 days), consistent with prior literature (26). Former and current use categories were combined into an ever-use category when aggregating transitions if necessary to protect participant confidentiality (see analyses).

#### 2.2.2 Adults

Among adults who smoked cigarettes, we evaluated transitions in cigarette smoking while considering the frequency of ENDS use. Participants were asked: “In the past 30 days, have you smoked a cigarette, even one or two puffs?” For the first interview wave in each wave triplicate (i.e., 2014 in the first period, 2014–2016–2017; 2019 in the second period, 2019–2021–2022), we created a three-level cigarette/ENDS use variable (regardless of other product use): (1) cigarettes only (smoked cigarettes in the past 30 days and did not use ENDS in the past 30 days), (2) cigarettes plus less frequent ENDS use (smoked cigarettes in the past 30 days and used ENDS on 1–19 days in the past 30 days), and (3) cigarettes plus frequent ENDS use (smoked cigarettes in the past 30 days and used ENDS on 20+ days in the past 30 days).

For the second interview wave in each wave triplicate, we created a six-level cigarette/ENDS use variable (regardless of other product use): (1) cigarettes only, (2) cigarettes plus less frequent ENDS use, (3) cigarettes plus frequent ENDS use, (4) neither product use (did not smoke cigarettes in past 30 days and did not use ENDS in past 30 days), (5) less frequent ENDS use only (did not smoke cigarettes in past 30 days and used ENDS 1–19 days in past 30 days), and (6) frequent ENDS use only (did not smoke cigarettes in past 30 days and used ENDS 20+ days in past 30 days).

For the third interview wave in each wave triplicate, we created a two-level cigarette smoking variable (regardless of ENDS use and other product use): (1) cigarette smoking (smoked cigarettes in the past 30 days), and (2) no cigarette smoking (did not smoke cigarettes in the past 30 days). Additionally, among adults, we created a four-level cigarette smoking and ENDS use (dual use) variable: (1) cigarette smoking only (smoked cigarettes in past 30 days and did not use ENDS in the past 30 days), (2) dual use (smoked cigarettes in past 30 days and used ENDS in the past 30 days), (3) neither product use (did not smoke cigarette in the past 30 days and did not use ENDS in the past 30 days), and (4) ENDS use only (did not smoke cigarettes in the past 30 days, did use ENDS in the past 30 days). This additional approach was used to assess dual use among adults at the final measurement.

### 2.3 Statistical analyses

#### 2.3.1 Trajectories in ENDS use among youth

We conducted two sets of analyses among youth: one set across the period 2014–2016–2017, and another set across the period 2019–2021–2022. Our first set of analyses was conducted among youth ages 12–17 in 2014. We evaluated transitions in ENDS use status between 2014 and 2016, and we evaluated transitions in ENDS use status between 2016 and 2017, conditional on transitions in ENDS use between 2014 and 2016. These analyses were conducted among all youth in 2014 and were stratified by ENDS use status in 2014. Our second set of analyses was conducted among youth ages 12–17 in 2019. We evaluated transitions in ENDS use status between 2019 and 2021, and we evaluated transitions in ENDS use status between 2021 and 2022, conditional on transitions in ENDS use between 2019 and 2021. These analyses were conducted among all youth in 2019 and were stratified by ENDS use status in 2019. See Supplementary Tables S3–S12 for delineation of how transitions were enumerated across waves among youth. See Supplementary Figures S1–S5 for visualization of how transitions were enumerated across waves among youth.

#### 2.3.2 Trajectories in cigarette smoking status among adults who smoked cigarettes

We also conducted two sets of analyses among adults who smoked cigarettes: one set across the period 2014–2016–2017, and the other across the period 2019–2021–2022. Our first set of analyses was conducted among adults ages 18+ who smoked cigarettes in 2014. We evaluated transitions in cigarette and ENDS use status between 2014 and 2016, and we evaluated transitions in cigarette use status between 2016 and 2017 conditional on transitions in cigarette and ENDS use between 2014 and 2016. Our second set of analyses was conducted among adults ages 18+ who smoked cigarettes in 2019. We evaluated transitions in cigarette and ENDS use status between 2019 and 2021, and we evaluated transitions in cigarette use status between 2021 and 2022, conditional on transitions in cigarette and ENDS use between 2019 and 2021. Among adults at the last measure, we evaluated the two-level cigarette smoking variable (regardless of ENDS use) and the four-level cigarette smoking and ENDS use variable (see “Measures” section). See Supplementary Tables S12–S16 for the delineation of how transitions were enumerated across waves among adults. See Supplementary Figures S6–S9 for visualization of how transitions were enumerated across waves among adults.

#### 2.3.3 Weighting

For both youth and adults, the analyses across the period 2014–2016–2017 were weighted using the PATH Study Wave 4 longitudinal sampling weights for the Wave 1 cohort, and the analyses for the period 2019–2021–2022 were weighted using the PATH Study Wave 7 longitudinal sampling weights for the Wave 4 cohort. All confidence intervals were computed using the balanced repeated replication (BRR) method (27) with Fay's adjustment (28) set to 0.3 to increase stability of estimates. Analyses were conducted using Stata software (RRID:SCR_012763).

## 3 Results

### 3.1 Population characteristics

In 2019, the youth population was 51% male (*n* = 3,730), 49% female (*n* = 3,576); 65% white ethnicity only (*n* = 4,572), 14% Black ethnicity only (*n* = 1,093), 20% other ethnicity including multi-ethnicity (*n* = 1,660); 24% Hispanic ethnicity (*n* = 2,162), and 76% not Hispanic ethnicity (*n* = 5,163; Supplementary Table S1). In 2019, the population of adults who smoked cigarettes was 54% male (*n* = 2,705), 46% female (*n* = 3,092); 74% white ethnicity only (*n* = 4,012), 16% Black ethnicity only (*n* = 1,092), 10% Other ethnicity including multi-ethnicity (*n* = 696); 14% Hispanic ethnicity (*n* = 931), 86% not Hispanic ethnicity (*n* = 4,869); 11% ages 18–24 (*n* = 1,010), 25% ages 25–34 (*n* = 1,455), 20% ages 35–44 (*n* = 967), 17% ages 45–54 (*n* = 948), and 27% ages 55 or older (*n* = 1,420; Supplementary Table S2).

### 3.2 Overview of ENDS use among youth

Among the estimated 23.4 million [95% confidence interval (CI): 23.1–23.7 million] youth in the US in 2014, 2.7% (95% CI: 2.6–2.7%) used ENDS frequently in 2017 (Table 1). Among the estimated 23.8 million (95% CI: 23.6–23.9 million) youth in the US in 2019, 6.7% (95% CI: 6.0–7.3%) used ENDS frequently in 2022 (Table 1).

**Table 1:** Overview of ENDS use among the US population of youth across the 2014–2016–2017 period and across the 2019–2021–2022 period.

**Population**		**Population of youth in 2014 (denominator)**			**Frequent ENDS use in 2017 (numerator, row percentages)**				
	**ENDS use status in 2014**	***n***	**Weighted** *n*^a^	**95% CI** ^a^	***n***	**Weighted** *n*^a^	**95% CI** ^a^	**%** ^a^	**95% CI** ^a^
Youth, 2014–2016–2017 period	Never used ENDS	8,747	21,219,000	[20,419,000–22,019,000]	142	412,000	[61,000–763,000]	1.9	[0.8, 4.4]
	Formerly used ENDS	609	1,581,000	[702,000–2,461,000]	45	115,000	[0–376,000]	7.3	[0.8, 44.2]
	Less frequently used ENDS	203	541,000	[0–1,203,000]	28	84,000	[0–253,000]	15.5	[4.6, 41.2]
	Frequently used ENDS	23	62,000	[0–253,000]	5	14,000	[0–94,000]	22.9	[1.8, 82.7]
	Overall	9,582	23,404,000	[23,061,000–23,747,000]	220	625,000	[617,000–632,000]	2.7	[2.6, 2.7]
**Population**		**Population of youth in 2019 (denominator)**			**Frequent ENDS use in 2022 (numerator, row percentages)**				
**ENDS use status in 2019**	***n***	**Weighted** *n*^b^	**95% CI** ^b^	**n**	**Weighted** *n*^b^	**95% CI** ^b^	**%** ^b^	**95% CI** ^b^
Youth, 2019–2021–2022 period	Never used ENDS	6,031	19,635,000	[19,373,000–19,896,000]	162	505,000	[423,000–588,000]	2.6	[2.2, 3.0]
	Formerly used ENDS	738	2,360,000	[2,175,000–2,545,000]	117	399,000	[319,000–478,000]	16.9	[13.9, 20.4]
	Less frequently used ENDS	424	1,317,000	[1,190,000–1,444,000]	119	392,000	[313,000–472,000]	29.8	[24.9, 35.2]
	Frequently use ENDS	132	442,000	[345,000–539,000]	89	286,000	[221,000–351,000]	64.8	[55.0, 73.4]
	Overall	7,325	23,753,000	[23,587,000–23,920,000]	487	1,582,000	[1,427,000–1,738,000]	6.7	[6.0, 7.3]

Unweighted Ns are shown. Weighted Ns and corresponding 95% CIs are shown rounded to the nearest 1,000. Weighted row percentages are shown.

The weighted denominators shown in this table can be used with the weighted percentage data shown in Supplementary Tables to calculate the estimated population sizes for each trajectory in ENDS use among youth in the US across the 2014–2016–2017 period and for each trajectory in ENDS use among youth in the US in the 2019–2021–2022 period (e.g., Supplementary Table S3, first row, 0.03% of this table population of 23,404,000 = 7,000 youth who used ENDS frequently in 2014, 2016, and 2017; Supplementary Table S4, first row, 0.94% of this table population of 23,753,000 = 223,000 youth who used ENDS frequently in 2019, 2021 and 2022).

^a^PATH Study Public Use Files were used. Estimates were weighted using the Wave 4 longitudinal sampling weights for the Wave 1 cohort.

^b^PATH Study Restricted Use Files were used. Estimates were weighted using the Wave 7 longitudinal sampling weights for the Wave 4 cohort.

### 3.3 Trajectories in ENDS use among youth

Aggregated trajectories in youths' ENDS use in the US across the two periods, 2014–2016–2017; 2019–2021–2022, are shown in Table 2, and details of all individual trajectories are shown in Supplementary material—as indicated in the Table 2 footnotes. Across the period 2014–2016–2017, 13% (95% CI: 1–69%) of youth who used ENDS frequently in 2014 also used ENDS frequently in 2016 and 2017; across the period 2019–2021–2022, 51% (95% CI: 42–60%) of youth who used ENDS frequently in 2019 also used ENDS frequently in 2021 and 2022 (Table 2).

**Table 2:** Aggregated trajectories in youths' ENDS use in the US across the 2014–2016–2017 period and across the 2019–2021–2022 period.

**Youth ENDS use, beginning of period**	**Aggregated trajectories across the period**	**Youth, 2014–2016–2017 period**					**Youth, 2019–2021–2022 period**				
		***n***	**Weighted *n*^a^**	**95% CI^a^**	**%^a^**	**95% CI^a^**	***n***	**Weighted *n*^b^**	**95%CI^b^**	**%^b^**	**95%CI^b^**
Never used ENDS	Initiated ever ENDS use at the interim and/or last measure	2,250	5,711,000	4,912,000–6,511,000	27%	24–30%	1,002	3,299,000	3,079,000–3,522,000	17%	16–18%
Formerly used ENDS	Returned to ENDS use in P30D at interim and/or last measure	238	641,000	221,000–1,061,000	41%	22–62%	270	906,000	790,000–1,021,000	38%	34–43%
	Returned to ENDS use in P30D at the interim and last measure	68	173,000	28,000–319,000	11%	4–27%	105	365,000	289,000–441,000	15%	13–19%
Used ENDS less frequently	Progressed to frequent ENDS use in P30D at interim and/or last measure	41	126,000	0–388,000	23%	8–51%	141	459,000	379,000–539,000	35%	30–40%
	Progressed to frequent ENDS use in P30D at the interim and last measure	10	32,000	0–92,000	6%	1–36%	61	218,000	154,000–282,000	17%	13–22%
Used ENDS frequently	Maintained frequent ENDS use in P30D at interim and/or last measure	8	25,000	0–112,000	40%	5–89%	93	298,000	230,000–366,000	67%	57–76%
	Maintained frequent ENDS use in P30D at the interim and last measure	3	8,000	0–33,000	13%	1–69%	69	224,000	169,000–279,000	51%	42–60%

Unweighted Ns are shown. Weighted Ns and corresponding 95% CIs are shown rounded to the nearest 1,000. Weighted cell percentages are shown. See Table 1 for denominators for each ENDS use status group. Individual trajectories in ENDS use among youth in the 2014–2016–2017 period are shown in Supplementary Figure S1A, and point estimates with confidence intervals for each trajectory are shown in Supplementary Table S3. Individual trajectories in ENDS use among youth in the 2014–2016–2017 period, stratified by ENDS use status in 2014, are shown in Supplementary Figures S2A–S5A, and point estimates with confidence intervals for each trajectory are shown in Supplementary Tables S5–S8.

Individual trajectories in ENDS use among youth in the 2019–2021–2022 period are shown in Supplementary Figure S1B, and point estimates with confidence intervals for each trajectory are shown in Supplementary Table S4. Individual trajectories in ENDS use among youth in the 2019–2021–2022 period, stratified by ENDS use status in 2019, are shown in Supplementary Figures S2B–S5B, and point estimates with confidence intervals for each trajectory are shown in Supplementary Tables S9–S12.

^a^PATH Study Public Use Files were used. Estimates were weighted using the Wave 4 longitudinal sampling weights for the Wave 1 cohort.

^b^PATH Study Restricted Use Files were used. Estimates were weighted using the Wave 7 longitudinal sampling weights for the Wave 4 cohort.

### 3.4 Overview of cigarette discontinuation among adults who smoked cigarettes

Among the estimated 627,000 adults (95% CI: 483,000–771,000) in the US who smoked cigarettes and used ENDS frequently in 2014, 20.0% (95% CI: 16.3–31.5%) discontinued cigarette smoking in 2017 (Table 3). Among the estimated 3.8 million (95% CI: 3.4–4.2 million) adults who smoked cigarettes and used ENDS frequently in 2019, 42.4% (95% CI: 37.0–47.9%) discontinued cigarette smoking in 2022 (Table 3).

**Table 3:** Overview of cigarette discontinuation among the US population of adults who smoked cigarettes across the 2014–2016–2017 period and across the 2019–2021–2022 period.

**Population**		**Population of adults who smoked cigarettes in 2014 (denominator)**			**No Cigarette smoking in 2017 (numerator, row percentages)**				
	**ENDS use status in 2014**	***n***	**Weighted** *n*^a^	**95% CI** ^a^	***n***	**Weighted** *n*^a^	**95% CI** ^a^	**%** ^a^	**95% CI** ^a^
Adults, 2014–2016–2017 period	Cigarettes only	6,755	38,287,000	[37,000,000–39,573,000]	1,240	7,221,000	[6,654,000–7,788,000]	18.9	[17.6, 20.2]
	Cigarettes+less frequent ENDS	2,128	11,407,000	[10,877,000–11,937,000]	416	2,300,000	[2,028,000–2,572,000]	20.2	[18.3, 22.2]
	Cigarettes+frequent ENDS	116	627,000	[483,000–771,000]	28	144,000	[88,000–201,000]	20.0	[16.3, 31.5]
	Overall	8,999	50,321,000	[48,876,000–51,765,000]	1,684	9,665,000	[9,053,000–10,278,000]	19.2	[18.2, 20.3]
**Population**		**Population of adults who smoked cigarettes in 2019 (denominator)**			**No cigarette smoking in 2022 (numerator, row percentages)**				
**ENDS use status in 2019**	***n***	**Weighted** *n*^b^	**95% CI** ^b^	***n***	**Weighted** *n*^b^	**95% CI** ^b^	**%** ^b^	**95% CI** ^b^
Adults, 2019–2021–2022 period	Cigarettes only	4,139	33,656,000	[32,469,000–34,843,000]	929	7,894,000	[7,170,000–8,617,000]	23.5	[21.8, 25.3]
	Cigarettes+less frequent ENDS	1,131	7,855,000	[7,286,000–8,423,000]	398	2,714,000	[2,369,000–3,059,000]	34.6	[31.1, 38.2]
	Cigarettes+frequent ENDS	530	3,778,000	[3,393,000–4,163,000]	234	1,601,000	[1,344,000–1,858,000]	42.4	[37.0, 47.9]
	Overall	5,800	45,289,000	[43,886,000–46,692,000]	1561	12,208,000	[11,412,000–13,005,000]	27.0	[25.6, 28.4]

Unweighted Ns are shown. Weighted Ns and corresponding 95% CIs are shown rounded to the nearest 1,000. Weighted row percentages are shown.

The weighted denominators shown in Table 2 can be used with the weighted percentage data shown in Supplementary Tables to calculate the estimated population sizes for each trajectory in cigarette smoking by ENDS use among adults who smoked cigarettes in the US in the 2014–2016–2017 period and for each trajectory in cigarette smoking by ENDS use among adults who smoked cigarettes in the US in the 2019–2021–2022 period.

^a^PATH Study Public Use Files were used. Estimates were weighted using the Wave 4 longitudinal sampling weights for the Wave 1 cohort.

^b^PATH Study Restricted Use Files were used. Estimates were weighted using the Wave 7 longitudinal sampling weights for the Wave 4 cohort.

### 3.5 Trajectories in cigarette discontinuation among adults who smoked cigarettes

Aggregated trajectories in adults' cigarette discontinuation rates in the US across the two periods, 2014–2016–2017; 2019–2021–2022, are shown in Table 4, and details of all individual trajectories are shown in Supplementary material—as indicated in the Table 4 footnotes. Across the period 2014–2016–2017, 14% (95% CI: 9–20%) of adults who smoked cigarettes and used ENDS frequently in 2014 were not smoking cigarettes in 2016 and 2017; across the period 2019–2021–2022, 30% (95% CI: 24–35%) of adults who smoked cigarettes and used ENDS frequently in 2019 were not smoking cigarettes in 2021 and 2022 (Table 4). See Supplementary Tables S15, S16 and Supplementary Figures S8, S9 for results when disaggregating cigarette smoking and ENDS use at the last measure.

**Table 4:** Aggregated trajectories in adults' cigarette discontinuation in the US across the 2014–2016–2017 period and across the 2019–2021–2022 period.

**Adult ENDS use status, beginning of period**	**Aggregated trajectories across the period**	**Adults, 2014–2016–2017 period**					**Adults, 2019–2021–2022 period**				
		***n***	**Weighted *n*^a^**	**95% CI^a^**	**%^a^**	**95% CI^a^**	***n***	**Weighted *n*^b^**	**95%CI^b^**	**%^b^**	**95% CI^b^**
Did not use ENDS	Stopped cigarette smoking at the interim and/or last measure	1,527	8,719,000	8,106,000–9,332,000	23%	21–24%	1,133	9,443,000	8,648,000–10,238,000	28%	26–30%
	Stopped cigarette smoking at the interim and last measure	791	4,732,000	4,273,000–5,192,000	12%	11–13%	623	5,395,000	4,800,000–5,990,000	16%	15–18%
Used ENDS less frequently	Stopped cigarette smoking at the interim and/or last measure	497	2,733,000	2,423,000–3,042,000	24%	22–26%	479	3,191,000	2,828,000–3,553,000	41%	37–44%
	Stopped cigarette smoking at the interim and last measure	243	1,368,000	1,122,000–1,615,000	12%	10–14%	269	1,760,000	1,482,000–2,039,000	22%	20–36%
Used ENDS frequently	Stopped cigarette smoking at the interim and/or last measure	32	166,000	105,000–228,000	26%	19–35%	280	1,905,000	1,639,000–2,171,000	50%	45–56%
	Stopped cigarette smoking at the interim and last measure	17	85,000	46,000–124,000	14%	9–20%	168	1,116,000	892,000–1,310,000	30%	24–35%

Unweighted Ns are shown. Weighted Ns and corresponding 95% CIs are shown rounded to the nearest 1,000. Weighted cell percentages are shown. See Table 2 for denominators for each ENDS use status group.

Individual trajectories in cigarette smoking among adults in the 2014–2016–2017 period are shown in Supplementary Figure S6, and point estimates with confidence intervals for each trajectory are shown in Supplementary Table S13.

Individual trajectories in cigarette smoking among adults in the 2019–2021–2022 period are shown in Supplementary Figure S7, and point estimates with confidence intervals for each trajectory are shown in Supplementary Table S14.

^a^PATH Study Public Use Files were used. Estimates were weighted using the Wave 4 longitudinal sampling weights for the Wave 1 cohort.

^b^PATH Study Restricted Use Files were used. Estimates were weighted using the Wave 7 longitudinal sampling weights for the Wave 4 cohort.

## 4 Discussion

Growth in the market share of salt-based nicotine e-liquids, the COVID-19 pandemic, and increasing tobacco regulations may have impacted trajectories in ENDS use and cigarette smoking in the US. Our longitudinal population-level findings indicate that an estimated 224,000 youth used ENDS frequently in 2019 and continued to do so in 2021 and 2022 (compared to ~8,000 youth who used ENDS frequently in 2014 and continued to do so in 2016 and 2017). An estimated 1.1 million adults smoked cigarettes and used ENDS frequently in 2019, and no longer smoked cigarettes in 2021 and 2022 (compared to ~85,000 adults who smoked cigarettes and used ENDS frequently in 2014, and no longer smoked cigarettes in 2016 and 2017). These findings are consistent with salt-based nicotine ENDS having greater potential for addictiveness among youth, and greater nicotine substitution potential among adults who smoke cigarettes, compared to free-base nicotine ENDS (8, 9).

The US Center for Tobacco Products uses an APPH standard when making tobacco product marketing authorization decisions, meaning that the potential population benefits of a product must be weighed against its potential population harms of the product (1). The findings from our study enable multiple approaches to quantifying a population-level youth vaping/adult cigarette quitting ratio. For example, across the period 2019–2021–2022, the number of youths in 2019 who used ENDS frequently in 2022 was 1.6 million (Table 1), while the number of adults who smoked cigarettes and used ENDS frequently in 2019 who stopped smoking cigarettes in 2022 was also 1.6 million (Table 3, compared to approximately four times more youth vaping frequently than adults quitting smoking when they vaped frequently across the period 2014–2016–2017). Other ways of quantifying the population-level youth vaping/adult cigarette quitting ratio can be derived from our study. However, it is important to note that our study does not include adults who never smoked cigarettes; thus, vaping among this group cannot be directly accounted for in a harm–benefit ratio derived from our findings.

Our findings on youth initiation of ENDS use—moving from never use to ever use—showed an opposite pattern from movement toward frequent use; across the period 2019–2021–2022, ~17% of youth who never used ENDS in 2019 ever used ENDS in 2021 or 2022, whereas ~27% of youth who never used ENDS in 2014 ever used ENDS in 2016 or 2017. These findings indicate that, since 2019, ENDS reached a smaller fraction of youth than prior to 2019. These findings are consistent with ENDS flavor restrictions (12, 13), changes in ENDS marketing practices (15), and Tobacco 21 laws (14) having their intended effects to reduce the appeal of ENDS to youth and to reduce youths' access to ENDS.

When considering findings among adults, prior studies have consistently found non-frequent ENDS use to be unrelated or negatively related to cigarette discontinuation (4, 29–31). Here, when we evaluated cigarette discontinuation over a long term and specifically since 2019, we found that 35% of adults who smoked cigarettes and used ENDS less frequently (less than 20 days out of 30 days) quit smoking cigarettes 3 years later compared to 24% of adults who did not use ENDS at all. It is possible that lower frequency ENDS use may lead to higher frequency ENDS use over the long term, which in turn may yield greater nicotine substitution and cigarette discontinuation. This new finding for less frequent ENDS use has population-level importance because non-frequent ENDS use is much more common than frequent ENDS use among adults who smoke cigarettes (though no ENDS use at all is still most common).

### 4.1 Limitations

These population-level findings document trajectories in product use occurring across the two periods, 2014–2016–2017; 2019–2021–2022, two time periods that align with before and after the shift in the US ENDS market from free-based nicotine formulations being predominant to salt-based nicotine formulations being predominant, though we did not directly assess whether nicotine formulation itself had a causal effect on product use behaviors. The 2019–2021–2022 period also overlapped with COVID-19. Additionally, there were differences in the PATH Study protocols used (18) and the timespans between interviews before and after 2019. Finally, the population of youth and the population of adults who smoked cigarettes may have differed before and since 2019, such that potential cohort effects may be reflected in our population-level findings in addition to potential period effects. Our observational findings should be interpreted as descriptive and context-setting, recognizing that some estimates are based on very small sample sizes and have wide confidence intervals, rather than as hypothesis-testing. Subsequent work can investigate whether ENDS use in 2019 or later (during the salt-based nicotine ENDS period) is causally related to cigarette discontinuation; prior work has shown that ENDS use in 2017 (prior to salt-based nicotine ENDS having a foothold on the market) was not causally related to cigarette discontinuation (32). Additionally, subsequent work can investigate whether the smoking discontinuation patterns observed here differ between those who smoke daily and those who smoke non-daily, and further research can investigate trajectories in ENDS use across the entire range of adults who use ENDS, including those who do not smoke cigarettes.

### 4.2 Implications

Over the 2013/14-2022/23 period that spanned rapid evolution in the ENDS market, including growth in popularity of salt-based nicotine ENDS, the COVID-19 pandemic, and state and federal ENDS restrictions and tobacco control policies, our findings indicate there are now greater rates of frequent ENDS use among youth alongside greater rates of cigarette discontinuation among adults who smoked cigarettes and used ENDS. Findings are consistent with the population-level impact of ENDS being greater across 2019–2021–2022 than across the period 2014–2016–2017.

## Data Availability

The data analyzed in this study is subject to the following licenses/restrictions: available via download or application. Requests to access these datasets should be directed to https://doi.org/10.3886/Series606.
