# Supplementary material for: Vaping and smoking trajectories among youth and adults in the United States across the period 2014–2022
Source: Front Public Health. 2026 Feb 23;14:1775015. doi: 10.3389/fpubh.2026.1775015 (PMC12968259; doi:10.3389/fpubh.2026.1775015)
Supplement: Supplementary file 1 [file Data_Sheet_1.pdf]

Supplementary Material

1 Table of Contents

1.1 Figures

1.1.1 Youth

SFigure 1A. Trajectories in youth ENDS use across 2014-2016-2017 (N=9,582)……..………**Page 3**

SFigure 1B. Trajectories in youth ENDS use across 2019-2021-2022 (N=7,325)………...……**Page 4**

SFigure 2A. Trajectories in youth ENDS use across 2014-2016-2017 among youth who never used ENDS in 2014 (n=8,747)……………………………….…………**Page 5**

SFigure 2B. Trajectories in youth ENDS use across 2019-2021-2022 among youth who never used ENDS in 2019 (n=6,031)…………………………….….……**Page 5**

SFigure 3A. Trajectories in youth ENDS use across 2014-2016-2017 among youth who formerly used ENDS in 2014 (n=609)……………….……………….………**Page 6**

SFigure 3B. Trajectories in youth ENDS use across 2019-2021-2022 among youth who formerly used ENDS in 2019 (n=738)…………………………….….…**Page 6**

SFigure 4A. Trajectories in youth ENDS use across 2014-2016-2017 among youth who used ENDS less frequently in 2014 (n=203)……………….………..…..…**Page 7**

SFigure 4B. Trajectories in youth ENDS use across 2019-2021-2022 among youth who used ENDS less frequently in 2019 (n=424)……………...……………..……**Page 7**

SFigure 5A. Trajectories in youth ENDS use across 2014-2016-2017 among youth who used ENDS frequently in 2014 (n=23)……………...…….…………..………**Page 8**

SFigure 5B. Trajectories in youth ENDS use across 2019-2021-2022 among youth who used ENDS frequently in 2019 (n=132)……...……….……………..……..**Page 8**

1.1.2 Adults

SFigure 6A. Trajectories in adult cigarette smoking across 2014-2016-2017 among adults who smoked cigarettes in 2014 (N=8,999)………….…………..………………**Page 9**

SFigure 6B. Trajectories in adult cigarette smoking across 2019-2021-2022 among adults who smoked cigarettes in 2019 (N=5,800)……...…………………….………**Page 10**

SFigure 7A. Dual use in 2017 among adults who smoked cigarettes in 2014 (N=8,999)…...…**Page 11**

SFigure 7B. Dual use in 2022 among adults who smoked cigarettes in 2019 (N=5,800)...……**Page 12**

1.2 Tables

1.2.1 Demographic characteristics

STable 1A. Demographic characteristics of the US population of youth……………………………**Page 13**

STable 1B. Demographic characteristics of the US population of adults who smoked cigarettes……………………………………………………………………..…**Page 14**

1.2.2 Youth

STable 2A. Trajectories in youth ENDS use across 2014-2016-2017 (N=9,582)…….………**Page 15**

STable 2B. Trajectories in youth ENDS use across 2019-2021-2022 (N=7,325)………………**Page 17**

STable 3A. Trajectories in youth ENDS use across 2014-2016-2017 among youth who never used ENDS in 2014 (n=8,747)……….………………………………….………**Page 19**

STable 3B. Trajectories in youth ENDS use across 2019-2021-2022 among youth who never used ENDS in 2019 (n=6,031)…….………………………...…….………**Page 20**

STable 4A. Trajectories in youth ENDS use across 2014-2016-2017 among youth who formerly used ENDS in 2014 (n=609)…….……………...…………….………**Page 21**

STable 4B. Trajectories in youth ENDS use across 2019-2021-2022 among youth who formerly used ENDS in 2019 (n=738)……………………………………….………**Page 21**

STable 5A. Trajectories in youth ENDS use across 2014-2016-2017 among youth who used ENDS less frequently in 2014 (n=203)…………………………...………**Page 22**

STable 5B. Trajectories in youth ENDS use across 2019-2021-2022 among youth who used ENDS less frequently in 2019 (n=424)……………………….……….………**Page 22**

STable 6A. Trajectories in youth ENDS use across 2014-2016-2017 among youth who used ENDS frequently in 2014 (n=23)………...……………………...………**Page 23**

STable 6B. Trajectories in youth ENDS use across 2019-2021-2022 among youth who used ENDS frequently in 2019 (n=132)……...……………………..….………**Page 23**

1.2.3 Adults

STable 7A. Trajectories in adult cigarette smoking across 2014-2016-2017 among adults who smoked cigarettes in 2014 (N=8,999)…………………………………….………**Page 24**

STable 7B. Trajectories in adult cigarette smoking across 2019-2021-2022 among adults who smoked cigarettes in 2019 (N=5,800)………….…………………………….………**Page 26**

STable 8A. Dual use in 2017 among adults who smoked cigarettes in 2014 (N=8,999)………**Page 28**

STable 8B. Dual use in 2022 among adults who smoked cigarettes in 2019 (N=5,800)……....**Page 29**

2 Supplementary Figures

2.1 Youth

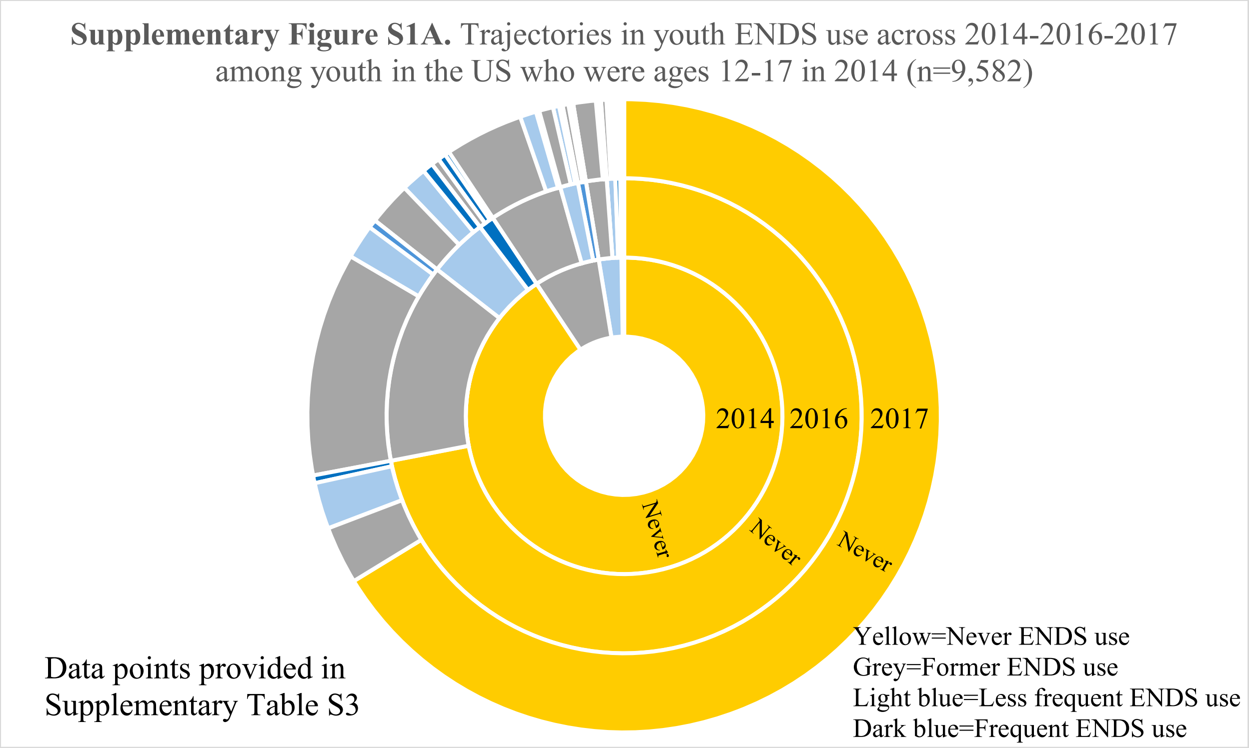

Supplementary Figure S1A. Trajectories in youth ENDS use across 2014-2016-2017 among youth in the US ages 12-17 in 2014. Inner ring indicates prevalence of ENDS use in 2014. Middle ring indicates transition rates between 2014-2016. Outer ring indicates transition rates between 2016-2017 contingent on transitions between 2014-2016.

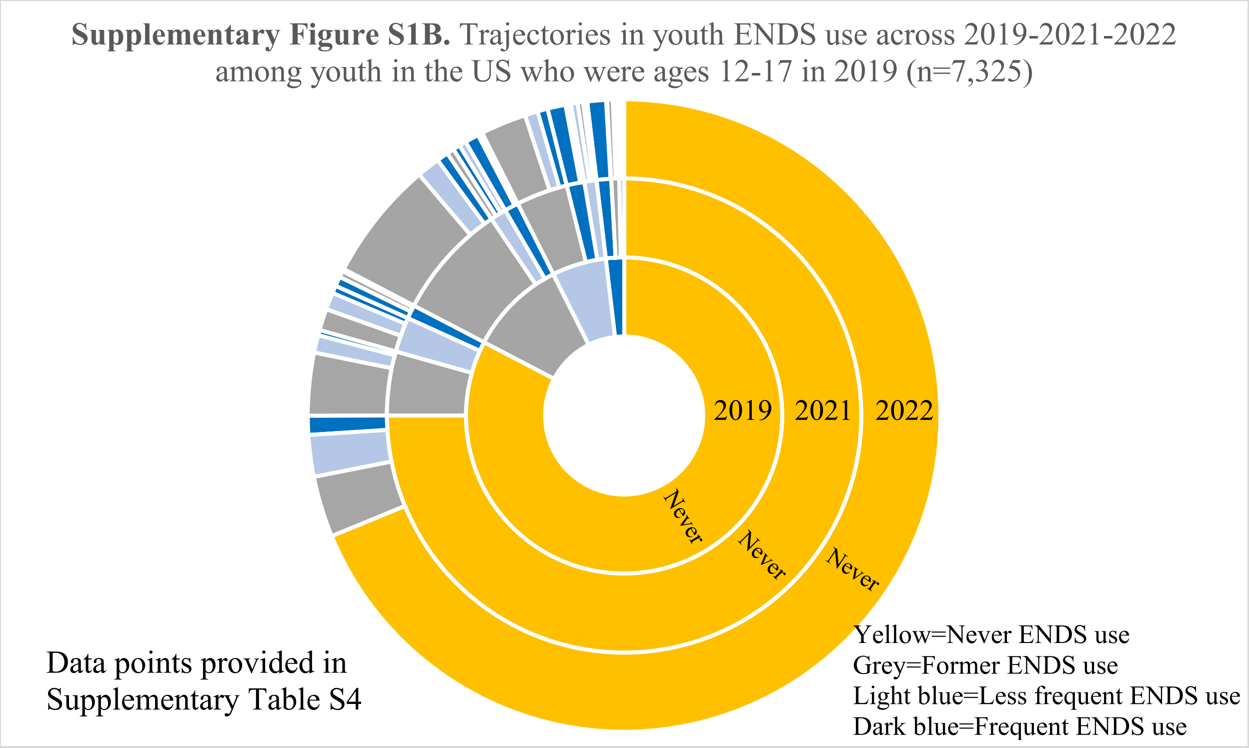

Supplementary Figure S1B. Trajectories in youth ENDS use across 2019-2021-2022 among youth in the US ages 12-17 in 2019. Inner ring indicates prevalence of ENDS use in 2019. Middle ring indicates transition rates between 2019-2021. Outer ring indicates transition rates between 2021-2022 contingent on transitions between 2019-2021.

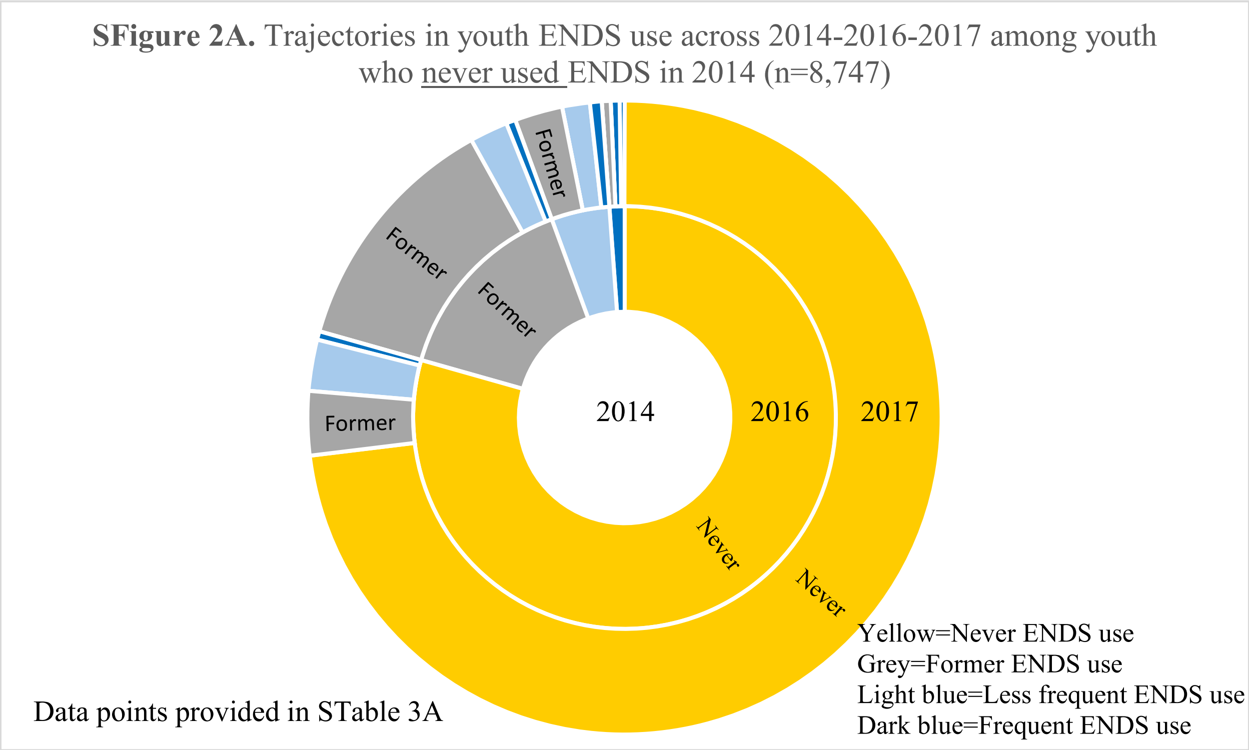

Supplementary Figure S2A. Trajectories in youth ENDS use across 2014-2016-2017 among youth who never used ENDS in 2014.

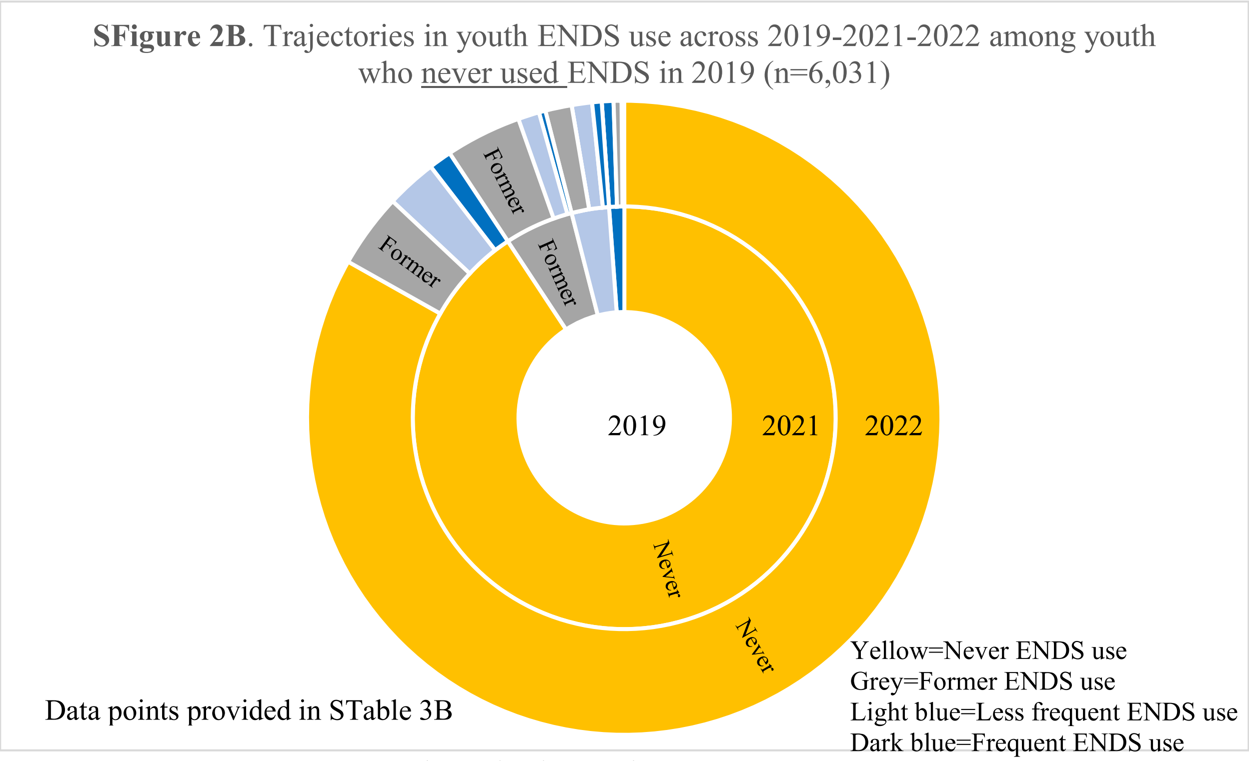

Supplementary Figure S2B. Trajectories in youth ENDS use across 2019-2021-2022 among youth who never used ENDS in 2019.

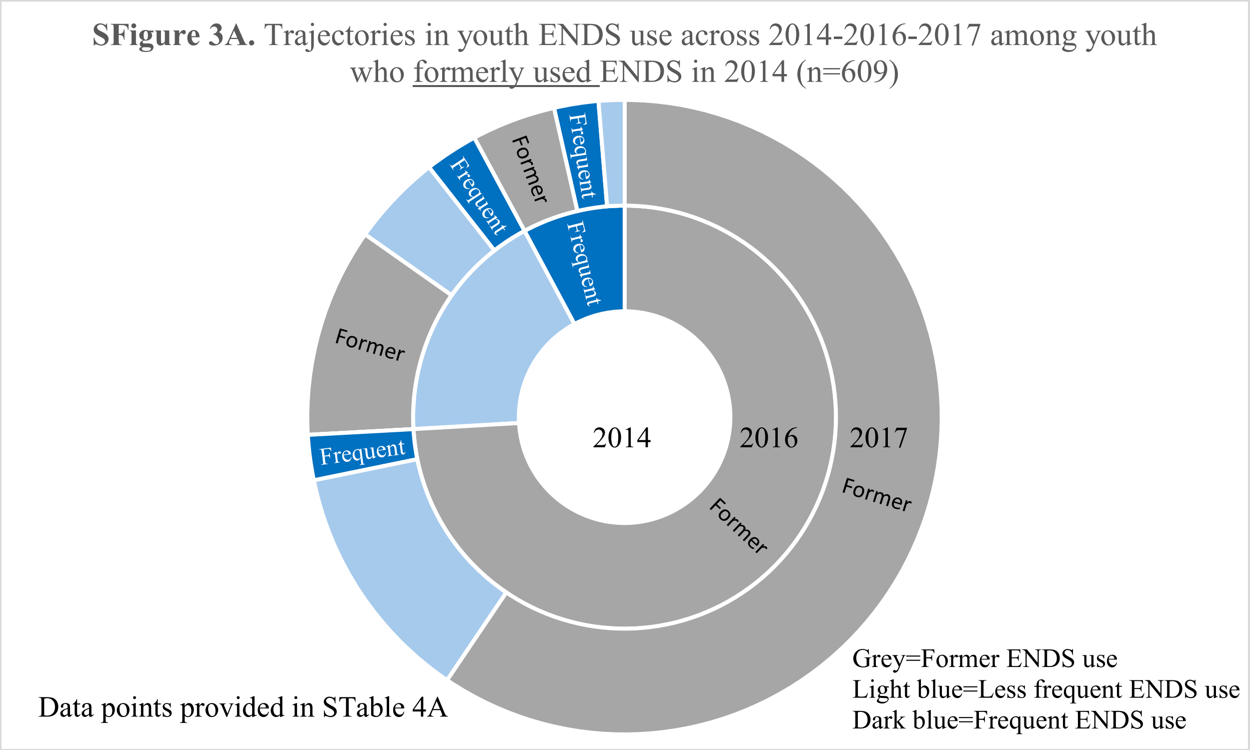

Supplementary Figure S3A. Trajectories in youth ENDS use across 2014-2016-2017 among youth who formerly used ENDS in 2014.

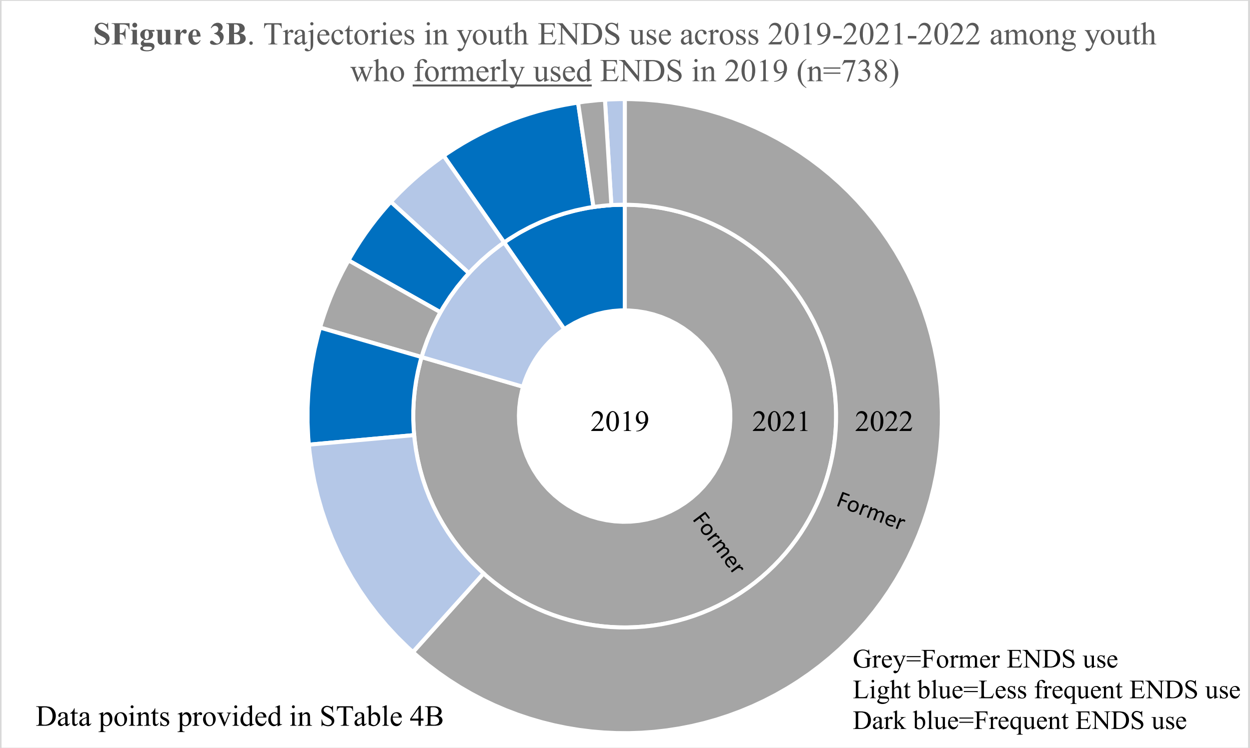

Supplementary Figure S3B. Trajectories in youth ENDS use across 2019-2021-2022 among youth who formerly used ENDS in 2019.

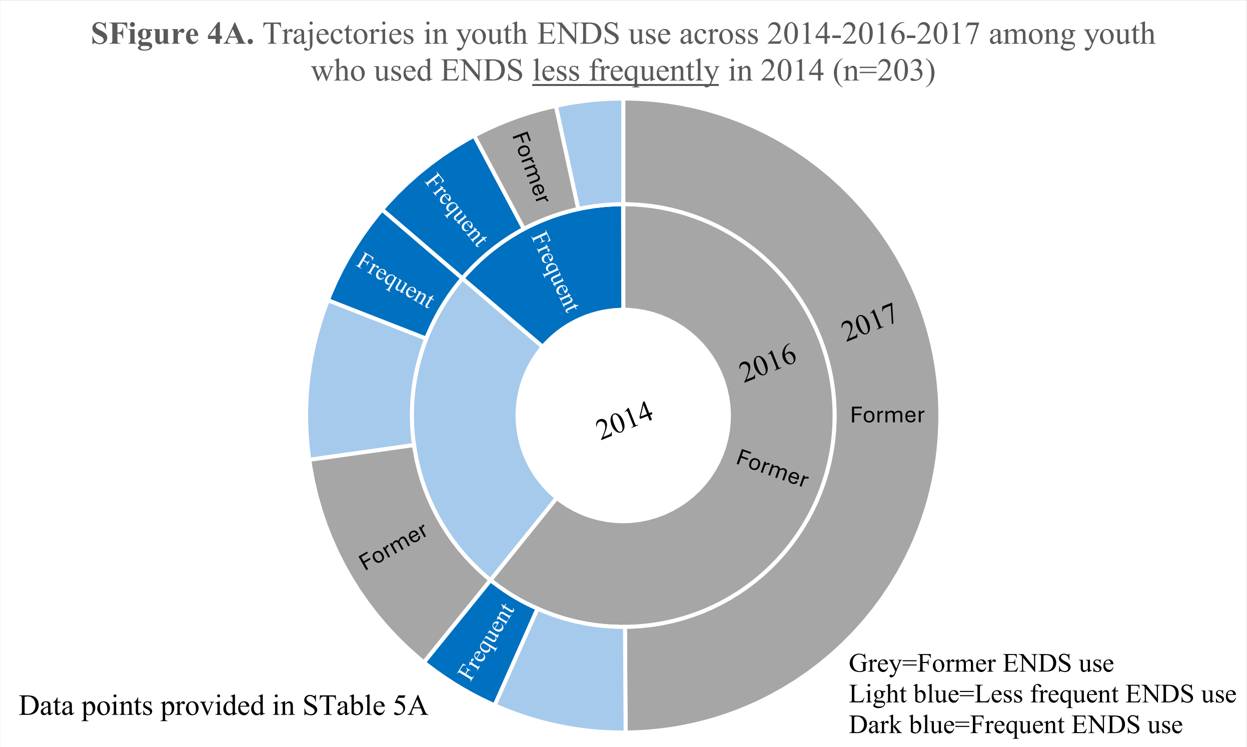

Supplementary Figure S4A. Trajectories in youth ENDS use across 2014-2016-2017 among youth who used ENDS less frequently in 2014.

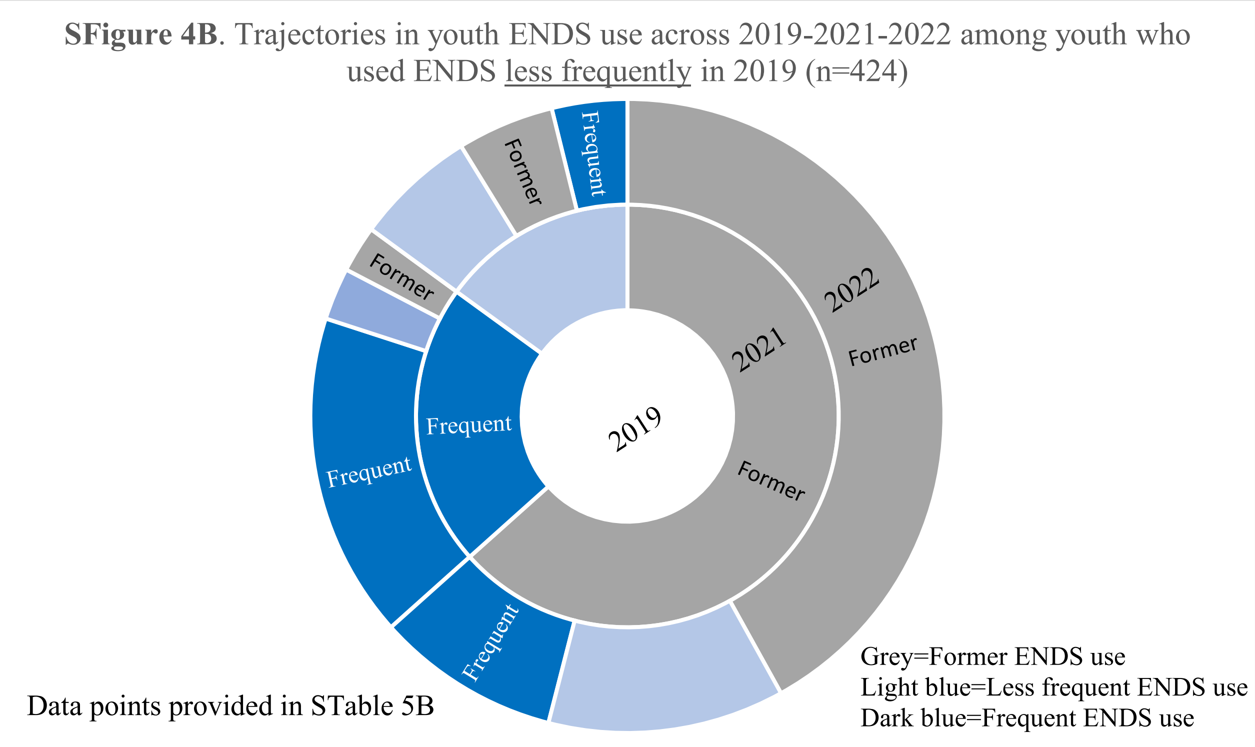

Supplementary Figure S4B. Trajectories in youth ENDS use across 2019-2021-2022 among youth who used ENDS less frequently in 2019.

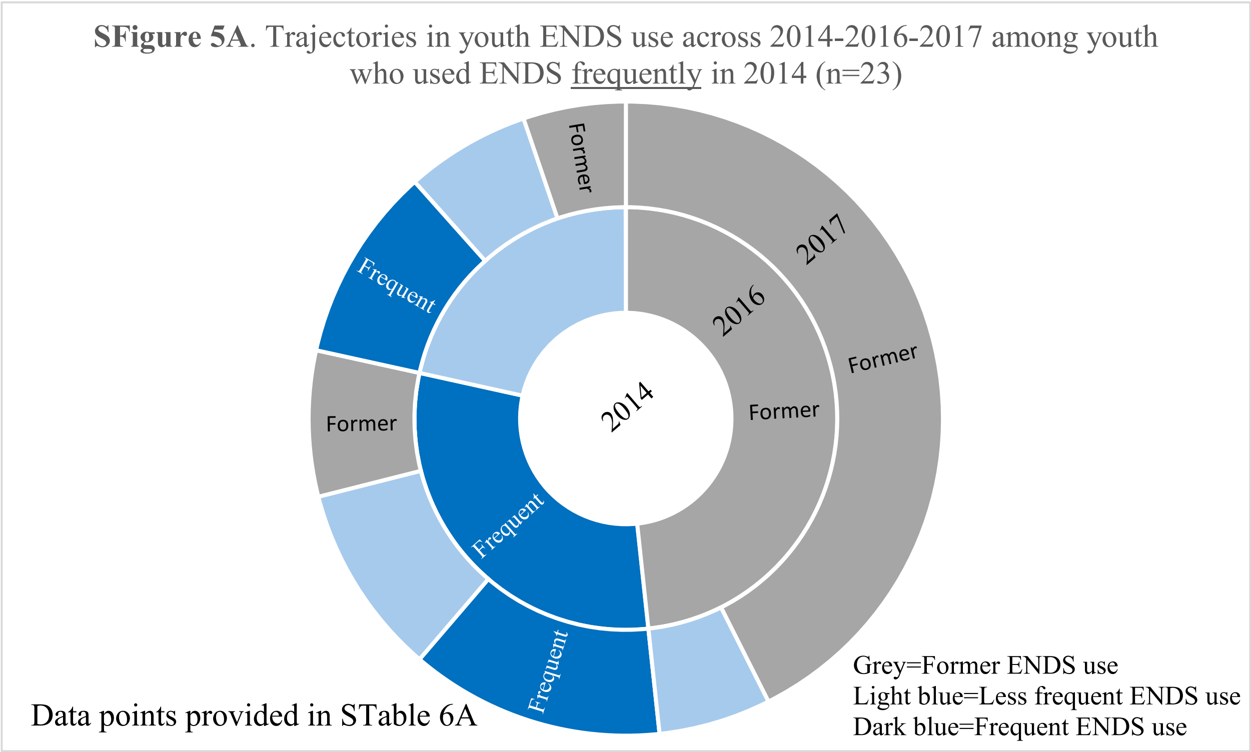

Supplementary Figure S5A. Trajectories in youth ENDS use across 2014-2016-2017 among youth who used ENDS frequently in 2014.

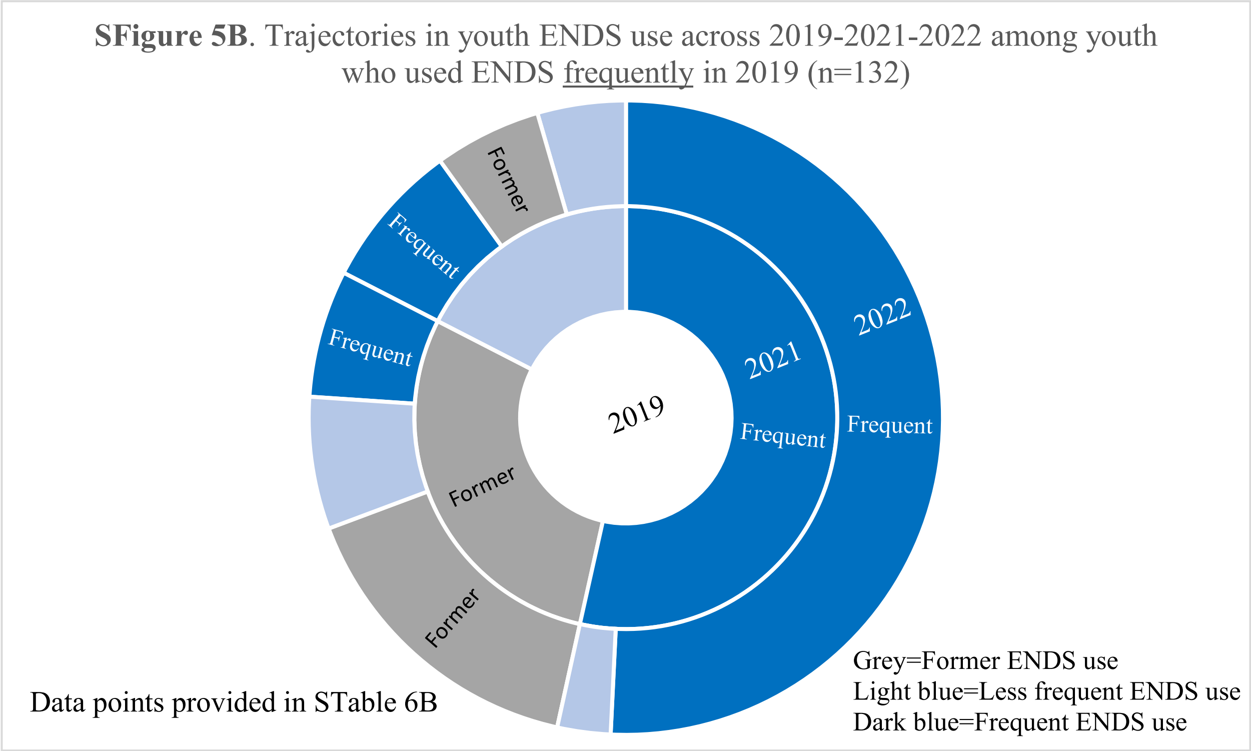

Supplementary Figure S5B. Trajectories in youth ENDS use across 2019-2021-2022 among youth who used ENDS frequently in 2019.

2.2 Adults

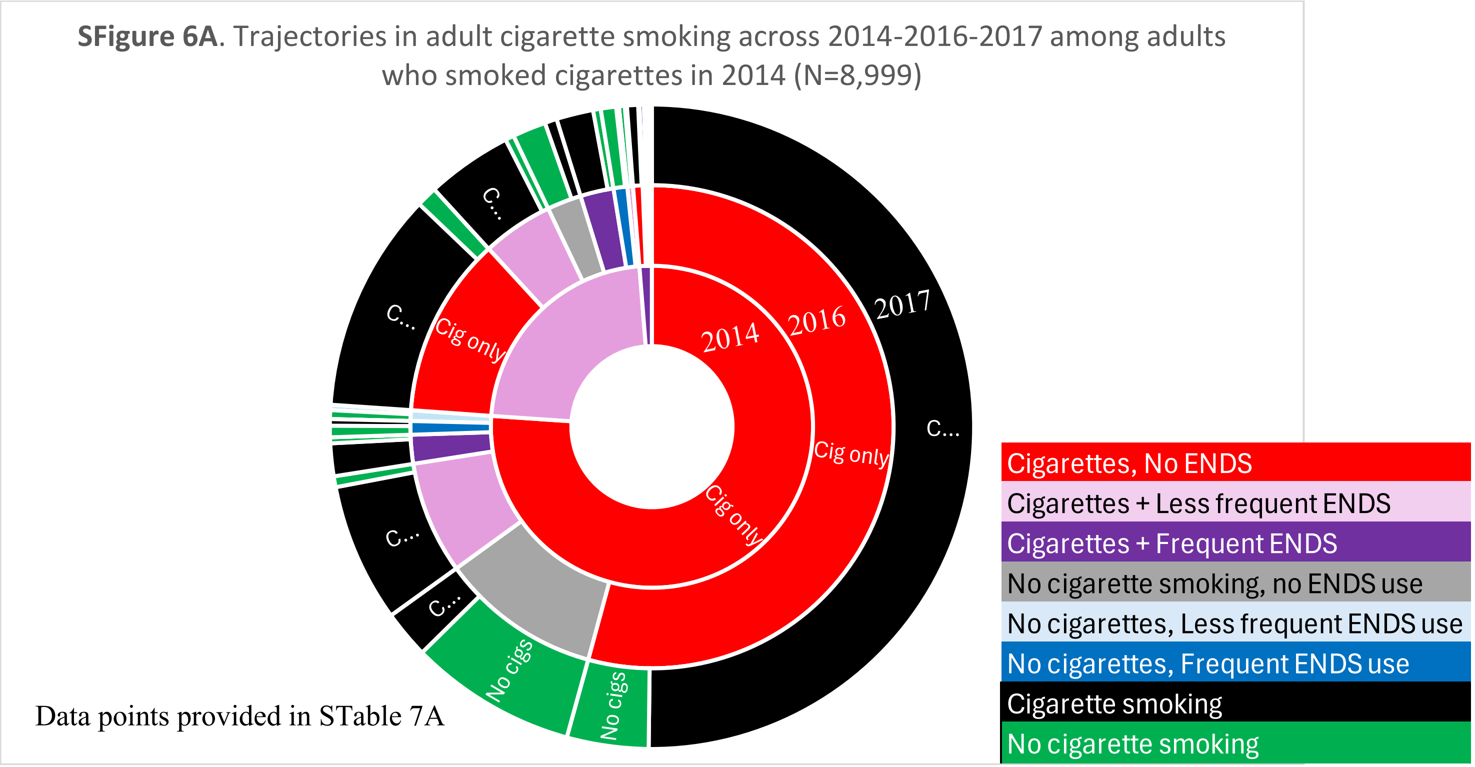

Supplementary Figure 6A. Trajectories in adult cigarette smoking across 2014-2016-2017 among adults who smoked cigarettes in 2014. Inner ring indicates prevalence of ENDS use among adults who smoked cigarettes in 2014. Middle ring indicates transition rates between 2014-2016. Outer ring indicates transition rates between 2016-2017 contingent on transitions between 2014-2016.

SFigure 6B. Trajectories in adult cigarette smoking across 2019-2021-2022 among adults smoked cigarettes in 2019 (N=5,800)

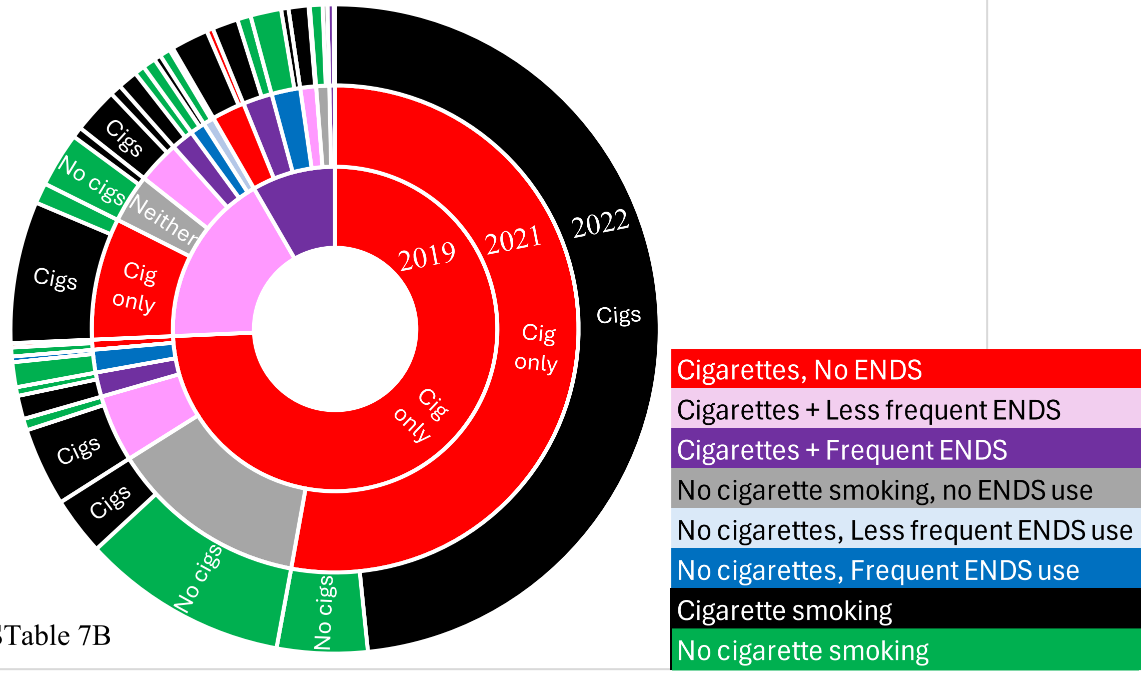

Data points provided in STable 7B

Supplementary Figure 6B. Trajectories in adult cigarette smoking across 2019-2021-2022 among adults who smoked cigarettes in 2019. Inner ring indicates prevalence of ENDS use among adult who smoked cigarettes in 2019. Middle ring indicates transition rates between 2019-2021. Outer ring indicates transition rates between 2021-2022 contingent on transitions between 2019-2021.

SFigure 7A. Dual use in 2017 among adults who smoked cigarettes in 2014 (N=8,999)

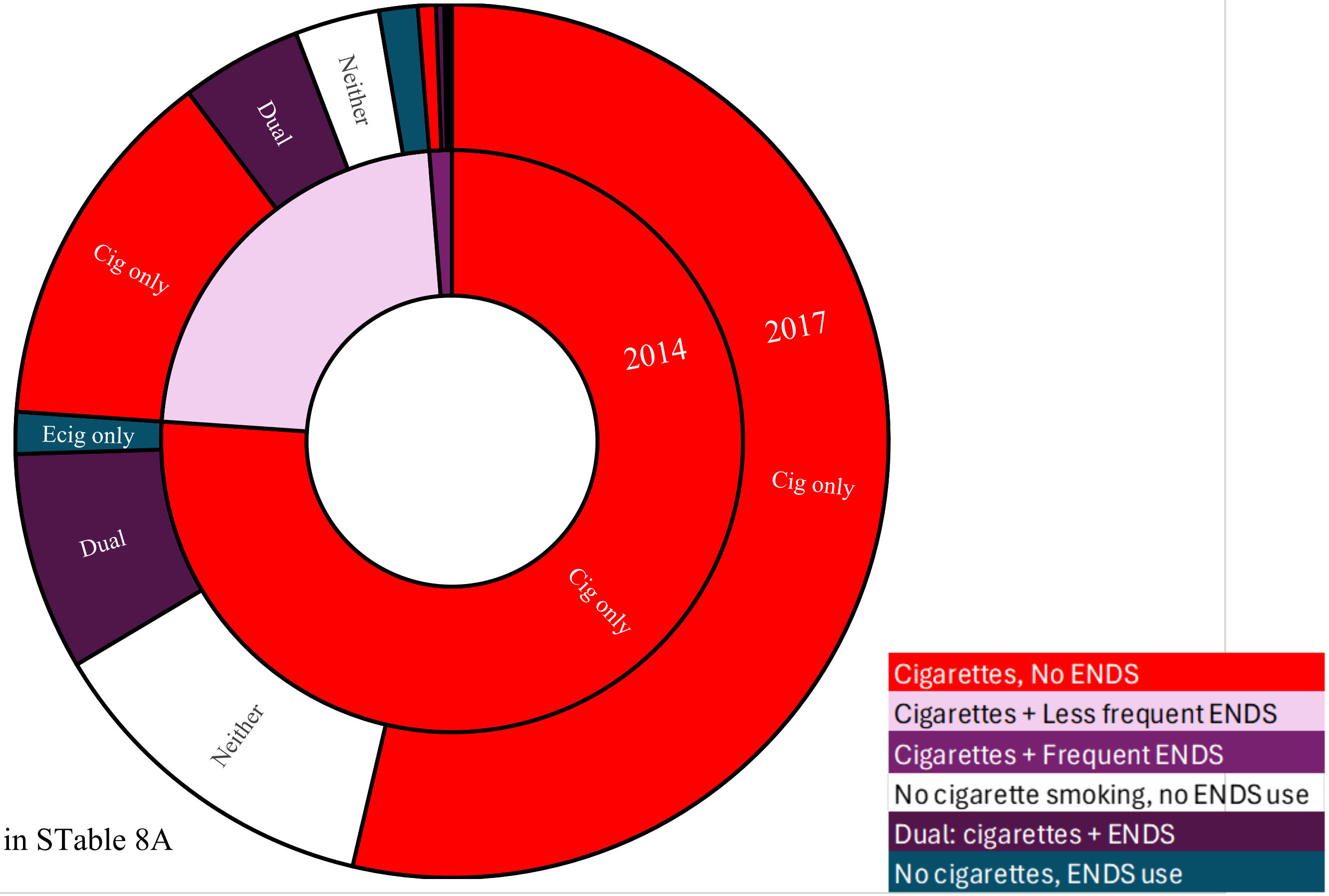

Data points provided in STable 8A

Supplemental Figure 7A. Dual use in 2017 among adults who smoked cigarettes in 2014. Inner ring indicates prevalence of ENDS use among adults who smoked cigarettes in 2014. Outer ring indicates dual use status in 2017 by use status in 2014.

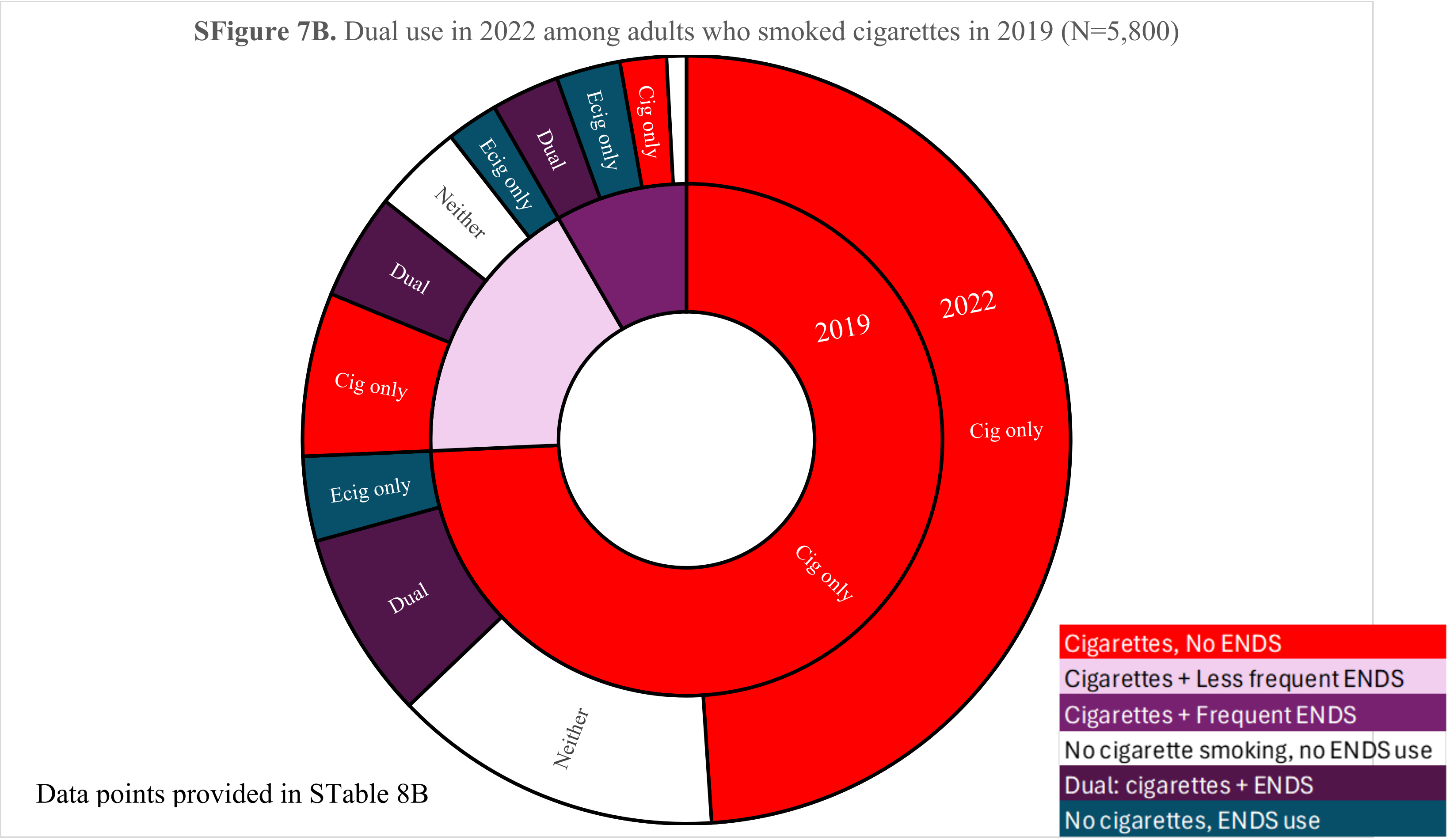

Supplemental Figure 7B. Dual use in 2022 among adults who smoked cigarettes in 2019. Inner ring indicates prevalence of ENDS use among adults who smoked cigarettes in 2019. Outer ring indicates dual use status in 2022 by use status in 2019.

3 Supplementary tables

3.1 Demographic characteristics

STable 1A. Demographic characteristics of the US population of youth.

		2014-2016-2017 period[1]		2019-2021-2022 period[2]	
		%	95%CI	%	95%CI
**Sex**	Male	51%	50%-52%	51%	51%-51%
	Female	49%	47%-51%	49%	49%-49%
**Age group**	12-14	52%	51%-53%	52%	51%-52%
	15-17	48%	47%-49%	48%	48%-49%
**Race**	White race only	71%	66%-75%	65%	64%-65%
	Black race only	15%	14%-17%	14%	14%-15%
	Other race including multi-racial	14%	9%-20%	20%	20%-22%
**Ethnicity**	Hispanic	22%	22%-22%	24%	24%-24%
	Not Hispanic	78%	78%-78%	76%	76%-76%

[1]PATH Study Public Use Files were used. N=9,582. Estimates were weighted using the Wave 4 longitudinal sampling weights for the Wave 1 cohort. Confidence intervals were computed using the balanced repeated replication (BRR) method with Fay's adjustment set to 0.3 to increase estimate stability. Column percentages are shown.
[2]PATH Study Restricted Use Files were used. N=7,325. Estimates were weighted using the Wave 7 longitudinal sampling weights for the Wave 4 cohort. Confidence intervals were computed using the balanced repeated replication (BRR) method with Fay's adjustment set to 0.3 to increase estimate stability. Column percentages are shown.

STable 1B. Demographic characteristics of the US population of adults who smoked cigarettes.

		2014-2016-2017 period[1]		2019-2021-2022 period[2]	
		%	95%CI	%	95%CI
**Sex**	Male	55%	54%-56%	54%	52%-55%
	Female	45%	44%-46%	46%	45%-48%
**Age group**	18-24	17%	16%-18%	11%	11%-12%
	25-34	24%	23%-25%	25%	24%-26%
	35-44	18%	17%-19%	20%	19%-22%
	45-54	19%	18%-20%	17%	16%-18%
	55+	22%	20%-23%	27%	25%-28%
**Race**	White race only	75%	74%-77%	74%	73%-75%
	Black race only	16%	15%-17%	16%	15%-17%
	Other race including multi-racial	8%	8%-9%	10%	9%-11%
**Ethnicity**	Hispanic	13%	13%-14%	14%	13%-15%
	Not Hispanic	87%	86%-87%	86%	85%-87%

[1]PATH Study Public Use Files were used. N=8,999. Estimates were weighted using the Wave 4 longitudinal sampling weights for the Wave 1 cohort. Confidence intervals were computed using the balanced repeated replication (BRR) method with Fay's adjustment set to 0.3 to increase estimate stability. Column percentages are shown.

[2]PATH Study Restricted Use Files were used. N=5,800. Estimates were weighted using the Wave 7 longitudinal sampling weights for the Wave 4 cohort. Confidence intervals were computed using the balanced repeated replication (BRR) method with Fay's adjustment set to 0.3 to increase estimate stability. Column percentages are shown.

3.2 Youth

STable 2A. Trajectories in youth ENDS use across 2014-2016-2017 (N=9,582).

ENDS use status					95% CI	
2014	2016	2017	n	%	lower bound	upper bound
Frequent	Frequent	Frequent	3	0.03	0.00	0.73
Frequent	Frequent	Less frequent	2	0.03	0.01	0.09
Frequent	Frequent	Former	1	0.02	0.00	93.48
Frequent	Less frequent	Frequent	2	0.03	0.00	94.20
Frequent	Less frequent	Less frequent	1	0.02	0.00	93.91
Frequent	Less frequent	Former	2	0.01	0.00	14.62
Frequent	Former	Frequent	0	0.00	-	-
Frequent	Former	Less frequent	1	0.02	0.00	98.13
Frequent	Former	Former	11	0.11	0.00	7.26
Less frequent	Frequent	Frequent	10	0.14	0.02	0.88
Less frequent	Frequent	Less frequent	6	0.08	0.00	6.36
Less frequent	Frequent	Former	7	0.10	0.00	2.74
Less frequent	Less frequent	Frequent	9	0.12	0.01	1.85
Less frequent	Less frequent	Less frequent	17	0.19	0.01	4.49
Less frequent	Less frequent	Former	25	0.28	0.01	5.17
Less frequent	Former	Frequent	9	0.10	0.00	2.31
Less frequent	Former	Less frequent	15	0.16	0.02	1.01
Less frequent	Former	Former	105	1.15	0.43	3.05
Former	Frequent	Frequent	14	0.15	0.00	9.77
Former	Frequent	Less frequent	7	0.09	0.00	41.26
Former	Frequent	Former	24	0.29	0.03	2.55
Former	Less frequent	Frequent	17	0.18	0.01	3.26
Former	Less frequent	Less frequent	30	0.32	0.05	2.05
Former	Less frequent	Former	63	0.72	0.09	5.31
Former	Former	Frequent	14	0.16	0.00	7.42
Former	Former	Less frequent	69	0.83	0.25	2.77
Former	Former	Former	371	4.02	1.87	8.43
Never	Frequent	Frequent	31	0.40	0.32	0.49

Never	Frequent	Less frequent	19	0.23	0.00	28.83
Never	Frequent	Former	36	0.41	0.05	3.00
Never	Less frequent	Frequent	42	0.55	0.11	2.77
Never	Less frequent	Less frequent	115	1.29	0.41	3.98
Never	Less frequent	Former	213	2.21	1.68	2.90
Never	Former	Frequent	36	0.43	0.05	3.48
Never	Former	Less frequent	153	1.75	0.47	6.31
Never	Former	Former	1092	11.42	9.61	13.53
Never	Never	Frequent	33	0.38	0.04	3.68
Never	Never	Less frequent	211	2.38	0.70	7.71
Never	Never	Former	269	2.96	1.19	7.14
Never	Never	Never	6497	66.26	63.15	69.24

Note. PATH Study Public Use Files were used. Ns are unweighted. Estimates were weighted using the PATH Study Wave 4 longitudinal sampling weights for the Wave 1 cohort. Confidence intervals were computed using the balanced repeated replication (BRR) method with Fay's adjustment set to 0.3 to increase estimate stability.

STable 2B. Trajectories in youth ENDS use across 2019-2021-2022 (N=7,325).

ENDS use status					95% CI	
2019	2021	2022	n	%	lower bound	upper bound
Frequent	Frequent	Frequent	69	0.94	0.74	1.21
Frequent	Frequent	Less freq+Former[1]	4	0.05	0.02	0.14
Frequent	Less frequent	Frequent	10	0.14	0.07	0.28
Frequent	Less frequent	Less frequent	7	0.08	0.04	0.19
Frequent	Less frequent	Former	7	0.10	0.04	0.27
Frequent	Former	Frequent	10	0.12	0.07	0.21
Frequent	Former	Less frequent	6	0.13	0.06	0.28
Frequent	Former	Former	19	0.29	0.18	0.50
Less frequent	Frequent	Frequent	61	0.92	0.68	1.23
Less frequent	Frequent	Less frequent	11	0.15	0.08	0.29
Less frequent	Frequent	Former	11	0.13	0.07	0.25
Less frequent	Less frequent	Frequent	18	0.21	0.12	0.38
Less frequent	Less frequent	Less frequent	26	0.34	0.22	0.54
Less frequent	Less frequent	Former	21	0.27	0.17	0.45
Less frequent	Former	Frequent	40	0.52	0.38	0.72
Less frequent	Former	Less frequent	52	0.67	0.50	0.90
Less frequent	Former	Former	184	2.33	2.01	2.69
Former	Frequent	Frequent	50	0.73	0.53	1.00
Former	Frequent	Less frequent	8	0.10	0.05	0.21
Former	Frequent	Former	12	0.13	0.07	0.26
Former	Less frequent	Frequent	22	0.36	0.24	0.54
Former	Less frequent	Less frequent	25	0.35	0.25	0.50
Former	Less frequent	Former	24	0.37	0.21	0.63
Former	Former	Frequent	45	0.59	0.43	0.81
Former	Former	Less frequent	84	1.19	0.93	1.51
Former	Former	Former	468	6.12	5.48	6.84
Never	Frequent	Frequent	35	0.50	0.36	0.69
Never	Frequent	Less frequent	8	0.12	0.06	0.22
Never	Frequent	Former	21	0.33	0.19	0.56

Never	Less frequent	Frequent	29	0.39	0.28	0.54
Never	Less frequent	Less frequent	61	0.87	0.64	1.17
Never	Less frequent	Former	74	1.11	0.85	1.45
Never	Former	Frequent	23	0.27	0.18	0.42
Never	Former	Less frequent	70	0.89	0.69	1.14
Never	Former	Former	219	3.20	2.75	3.72
Never	Never	Frequent	75	0.97	0.75	1.25
Never	Never	Less frequent	165	2.14	1.80	2.55
Never	Never	Former	222	3.11	2.65	3.65
Never	Never	Never	5029	68.77	67.55	69.96

Note. PATH Study Restricted Use Files were used. Ns are unweighted. Estimates were weighted using the PATH Study Wave 7 longitudinal sampling weights for the Wave 4 cohort. Confidence intervals were computed using the balanced repeated replication (BRR) method with Fay's adjustment set to 0.3 to increase estimate stability. [1]Less frequent and former groups were combined for failure to meet the threshold for dissemination separately.

STable 3A. Trajectories in youth ENDS use across 2014-2016-2017 among youth who never used ENDS in 2014 (n=8,747).

ENDS use status				95% CI	
2016	2017	n	%	lower bound	upper bound
Frequent	Frequent	31	0.44	0.36	0.53
Frequent	Less frequent	19	0.26	0.00	30.55
Frequent	Former	36	0.45	0.06	3.23
Less frequent	Frequent	42	0.60	0.12	3.05
Less frequent	Less frequent	115	1.42	0.46	4.28
Less frequent	Former	213	2.44	1.85	3.22
Former	Frequent	36	0.48	0.06	3.78
Former	Less frequent	153	1.93	0.51	6.98
Former	Former	1092	12.60	10.46	15.09
Never	Frequent	33	0.42	0.04	3.96
Never	Less frequent	211	2.62	0.79	8.32
Never	Former	269	3.26	1.28	8.06
Never	Never	6497	73.08	69.85	76.09

Note. PATH Study Public Use Files used. Ns unweighted. Estimates were weighted using PATH Study Wave 4 longitudinal sampling weights for the Wave 1 cohort. Confidence intervals computed using the BRR method.

STable 3B. Trajectories in youth ENDS use across 2019-2021-2022 among youth who never used ENDS in 2019 (n=6,031)**.**

ENDS use status				95% CI	
2021	2022	n	%	lower bound	upper bound
Frequent	Frequent	35	0.60	0.43	0.83
Frequent	Less frequent	8	0.14	0.07	0.27
Frequent	Former	21	0.40	0.23	0.68
Less frequent	Frequent	29	0.47	0.34	0.66
Less frequent	Less frequent	61	1.05	0.78	1.41
Less frequent	Former	74	1.34	1.03	1.75
Former	Frequent	23	0.33	0.21	0.51
Former	Less frequent	70	1.07	0.84	1.37
Former	Former	219	3.87	3.33	4.50
Never	Frequent	75	1.17	0.90	1.51
Never	Less frequent	165	2.59	2.18	3.08
Never	Former	222	3.76	3.20	4.42
Never	Never	5029	83.20	82.06	84.28

Note. PATH Study Restricted Use Files used. Ns unweighted. Estimates were weighted using PATH Study Wave 7 longitudinal sampling weights for the Wave 4 cohort. Confidence intervals computed using the BRR method.

STable 4A. Trajectories in youth ENDS use across 2014-2016-2017 among youth who formerly used ENDS in 2014 (n=609).

ENDS use status				95% CI	
2016	2017	n	%	lower bound	upper bound
Frequent	Frequent	14	2.27	0.02	74.39
Frequent	Less frequent	7	1.31	0.00	94.85
Frequent	Former	24	4.28	0.36	35.51
Less frequent	Frequent	17	2.71	0.17	30.64
Less frequent	Less frequent	30	4.68	1.08	18.04
Less frequent	Former	63	10.66	1.80	43.79
Former	Frequent	14	2.30	0.06	46.86
Former	Less frequent	69	12.35	2.29	45.85
Former	Former	371	59.45	37.80	77.96

Note. PATH Study Public Use Files used. Ns unweighted. Estimates were weighted using PATH Study Wave 4 longitudinal sampling weights for the Wave 1 cohort. Confidence intervals computed using the BRR method.

STable 4B. Trajectories in youth ENDS use across 2019-2021-2022 among youth who formerly used ENDS in 2019 (n=738).

ENDS use status				95% CI	
2021	2022	n	%	lower bound	upper bound
Frequent	Frequent	50	7.31	5.24	10.12
Frequent	Less frequent	8	1.00	0.49	2.03
Frequent	Former	12	1.35	0.70	2.61
Less frequent	Frequent	22	3.63	2.45	5.36
Less frequent	Less frequent	25	3.53	2.48	5.01
Less frequent	Former	24	3.67	2.12	6.30
Former	Frequent	45	5.94	4.38	8.01
Former	Less frequent	84	11.93	9.40	15.04
Former	Former	468	61.63	57.25	65.83

Note. PATH Study Restricted Use Files used. Ns unweighted. Estimates were weighted using PATH Study Wave 7 longitudinal sampling weights for the Wave 4 cohort. Confidence intervals computed using the BRR method.

STable 5A. Trajectories in youth ENDS use across 2014-2016-2017 among youth who used ENDS less frequently in 2014 (n=203).

ENDS use status				95% CI	
2016	2017	n	%	lower bound	upper bound
Frequent	Frequent	10	5.98	0.72	35.63
Frequent	Less frequent	6	3.42	0.09	56.89
Frequent	Former	7	4.36	0.19	52.19
Less frequent	Frequent	9	5.32	0.98	24.26
Less frequent	Less frequent	17	8.16	0.18	81.66
Less frequent	Former	25	11.96	1.53	54.34
Former	Frequent	9	4.16	0.38	33.30
Former	Less frequent	15	6.77	0.35	60.24
Former	Former	105	49.88	35.81	63.96

Note. PATH Study Public Use Files used. Ns unweighted. Estimates were weighted using PATH Study Wave 4 longitudinal sampling weights for the Wave 1 cohort. Confidence intervals computed using the BRR method.

STable 5B. Trajectories in youth ENDS use across 2019-2021-2022 among youth who used ENDS less frequently in 2019 (n=424).

ENDS use status				95% CI	
2021	2022	n	%	lower bound	upper bound
Frequent	Frequent	61	16.57	12.52	21.59
Frequent	Less frequent	11	2.67	1.37	5.15
Frequent	Former	11	2.38	1.22	4.58
Less frequent	Frequent	18	3.85	2.19	6.66
Less frequent	Less frequent	26	6.20	4.02	9.45
Less frequent	Former	21	4.94	3.10	7.79
Former	Frequent	40	9.39	6.87	12.71
Former	Less frequent	52	12.06	9.10	15.81
Former	Former	184	41.95	36.68	47.41

Note. PATH Study Restricted Use Files used. Ns unweighted. Estimates were weighted using PATH Study Wave 7 longitudinal sampling weights for the Wave 4 cohort. Confidence intervals computed using the BRR method.

STable 6A. Trajectories in youth ENDS use across 2014-2016-2017 among youth who used ENDS frequently in 2014 (n=23).

ENDS use status				95% CI	
2016	2017	n	%	lower bound	upper bound
Frequent	Frequent	3	12.98	1.01	68.67
Frequent	Less frequent	2	9.75	1.17	49.62
Frequent	Former	1	7.43	0.00	99.98
Less frequent	Frequent	2	9.94	0.01	99.57
Less frequent	Less frequent	1	6.37	0.00	99.98
Less frequent	Former	2	5.23	0.08	79.87
Former	Less frequent	1	5.73	0.00	99.99
Former	Former	11	42.57	2.90	94.85

Note. PATH Study Public Use Files used. Ns unweighted. Estimates were weighted using PATH Study Wave 4 longitudinal sampling weights for the Wave 1 cohort. Confidence intervals computed using the BRR method.

STable 6B. Trajectories in youth ENDS use across 2019-2021-2022 among youth who used ENDS frequently in 2019 (n=132).

ENDS use status				95% CI	
2021	2022	n	%	lower bound	upper bound
Frequent	Frequent	69	50.77	41.61	59.88
Frequent	Less freq+Former[1]	4	2.70		
Less frequent	Frequent	10	7.49	3.83	14.12
Less frequent	Less frequent	7	4.50	2.05	9.57
Less frequent	Former	7	5.47	2.03	13.91
Former	Frequent	10	6.49	3.68	11.18
Former	Less frequent	6	6.73	3.07	14.16
Former	Former	19	15.85	10.20	23.78

Note. PATH Study Restricted Use Files used. Ns unweighted. Estimates were weighted using PATH Study Wave 7 longitudinal sampling weights for the Wave 4 cohort. Confidence intervals computed using the BRR method.

[1]Less frequent and former groups were combined for failure to meet the threshold for dissemination separately.

3.3 Adults

STable 7A. Trajectories in adult cigarette smoking across 2014-2016-2017 among adults who smoked cigarettes in 2014 (N=8,999).

Cigarette smoking status					95% CI	
2014	2016	2017	n	%	lower bound	upper bound
Cig only	Cig only	Cigs	4440	50.16	48.88	51.44
Cig only	Cig only	No cigs	365	4.09	3.70	4.53
Cig only	Cig+Less freq	Cigs	646	6.96	6.46	7.49
Cig only	Cig+Less freq	No cigs	57	0.55	0.41	0.75
Cig only	Cig+freq	Cigs	142	1.64	1.35	1.98
Cig only	Cig+freq	No cigs	27	0.30	0.19	0.47
Cig only	Neither	Cigs	232	2.39	2.08	2.75
Cig only	Neither	No cigs	697	8.38	7.61	9.22
Cig only	Less freq only	Cigs	28	0.26	0.17	0.39
Cig only	Less freq only	No cigs	45	0.45	0.32	0.64
Cig only	Freq only	Cigs	27	0.33	0.21	0.52
Cig only	Freq only	No cigs	49	0.57	0.42	0.77
Cig+Less freq	Cig only	Cigs	1044	11.07	10.36	11.83
Cig+Less freq	Cig only	No cigs	100	1.02	0.82	1.28
Cig+Less freq	Cig+Less freq	Cigs	421	4.32	3.88	4.81
Cig+Less freq	Cig+Less freq	No cigs	39	0.44	0.31	0.63
Cig+Less freq	Cig+freq	Cigs	166	1.85	1.54	2.21
Cig+Less freq	Cig+freq	No cigs	34	0.39	0.27	0.58
Cig+Less freq	Neither	Cigs	58	0.61	0.45	0.82
Cig+Less freq	Neither	No cigs	158	1.67	1.39	2.01
Cig+Less freq	Less freq only	Cigs	11	0.10	0.05	0.20
Cig+Less freq	Less freq only	No cigs	27	0.28	0.18	0.46
Cig+Less freq	Freq only	Cigs	12	0.15	0.07	0.29
Cig+Less freq	Freq only	No cigs	58	0.76	0.56	1.05
Cig+freq	Cig only	Cigs	51	0.57	0.42	0.77
Cig+freq	Cig only	No cigs	4	0.06	0.02	0.17
Cig+freq	Cig+Less freq	Cigs	24	0.23	0.15	0.36

Cig+freq	Cig+Less freq	No cigs	5	0.05	0.03	0.09
Cig+freq	Cig+freq	Cigs	9	0.12	0.05	0.28
Cig+freq	Cig+freq	No cigs	2	0.01	0.00	0.05
Cig+freq	Neither	Cigs	1	0.01	0.00	0.06
Cig+freq	Neither	No cigs	9	0.08	0.04	0.16
Cig+freq	Less freq only	Cigs	2	0.02	0.00	0.12
Cig+freq	Less freq only	No cigs	1	0.01	0.00	0.06
Cig+freq	Freq only	Cigs	1	0.01	0.00	0.08
Cig+freq	Freq only	No cigs	7	0.08	0.04	0.15

Note. PATH Study Public Use Files used. Ns unweighted. Estimates were weighted using PATH Study Wave 4 longitudinal sampling weights for the Wave 1 cohort. Confidence intervals computed using the BRR method.

STable 7B. Trajectories in adult cigarette smoking across 2019-2021-2022 among adults who smoked cigarettes in 2019 (N=5,800).

Cigarette smoking status					95% CI	
2019	2021	2022	n	%	lower bound	upper bound
Cig only	Cig only	Cigs	2704	48.39	46.84	49.94
Cig only	Cig only	No cigs	241	4.51	3.77	5.39
Cig only	Cig+Less freq	Cigs	229	3.91	3.32	4.60
Cig only	Cig+Less freq	No cigs	35	0.57	0.38	0.83
Cig only	Cig+freq	Cigs	73	1.17	0.89	1.53
Cig only	Cig+freq	No cigs	30	0.44	0.28	0.68
Cig only	Neither	Cigs	173	2.90	2.41	3.49
Cig only	Neither	No cigs	520	10.24	9.19	11.41
Cig only	Less freq only	Cigs	14	0.23	0.13	0.42
Cig only	Less freq only	No cigs	31	0.44	0.30	0.65
Cig only	Freq only	Cigs	17	0.29	0.15	0.53
Cig only	Freq only	No cigs	72	1.23	0.90	1.67
Cig+Less freq	Cig only	Cigs	434	7.04	6.34	7.82
Cig+Less freq	Cig only	No cigs	58	1.04	0.75	1.42
Cig+Less freq	Cig+Less freq	Cigs	155	2.27	1.86	2.76
Cig+Less freq	Cig+Less freq	No cigs	38	0.56	0.38	0.82
Cig+Less freq	Cig+freq	Cigs	63	0.99	0.73	1.34
Cig+Less freq	Cig+freq	No cigs	33	0.51	0.34	0.76
Cig+Less freq	Neither	Cigs	42	0.54	0.36	0.80
Cig+Less freq	Neither	No cigs	176	2.70	2.21	3.28
Cig+Less freq	Less freq only	Cigs	13	0.18	0.08	0.40
Cig+Less freq	Less freq only	No cigs	43	0.54	0.38	0.78
Cig+Less freq	Freq only	Cigs	26	0.34	0.23	0.50
Cig+Less freq	Freq only	No cigs	50	0.65	0.46	0.91
Cig+freq	Cig only	Cigs	114	1.87	1.46	2.39
Cig+freq	Cig only	No cigs	19	0.32	0.20	0.53
Cig+freq	Cig+Less freq	Cigs	61	0.99	0.70	1.41
Cig+freq	Cig+Less freq	No cigs	4	0.07	0.02	0.22
Cig+freq	Cig+freq	Cigs	75	1.28	0.96	1.69

Cig+freq	Cig+freq	No cigs	43	0.68	0.47	0.97
Cig+freq	Neither	Cigs	15	0.23	0.14	0.39
Cig+freq	Neither	No cigs	42	0.63	0.40	1.00
Cig+freq	Less freq only	Cigs	6	0.07	0.03	0.18
Cig+freq	Less freq only	No cigs	19	0.31	0.16	0.60
Cig+freq	Freq only	Cigs	25	0.37	0.23	0.59
Cig+freq	Freq only	No cigs	107	1.52	1.20	1.93

STable 8A. Dual use in 2017 among adults who smoked cigarettes in pre-salt nicotine period (N=8,999).

Cigarette smoking status				95% CI	
2014	2017	n	%	lower bound	upper bound
Cig only	Cig only	4745	53.66	52.34	54.97
Cig only	Dual	770	8.08	7.45	8.75
Cig only	Neither	1094	12.80	11.84	13.82
Cig only	Ecig only	146	1.55	1.30	1.85
Cig+nonfreq	Cig only	1269	13.61	12.84	14.40
Cig+nonfreq	Dual	443	4.49	4.09	4.94
Cig+nonfreq	Neither	293	3.13	2.74	3.57
Cig+nonfreq	Ecig only	123	1.44	1.16	1.79
Cig+freq	Cig only	62	0.66	0.50	0.87
Cig+freq	Dual	26	0.30	0.19	0.47
Cig+freq	Neither	15	0.14	0.08	0.24
Cig+freq	Ecig only	13	0.15	0.09	0.25

Note. PATH Study Public Use Files used. Ns unweighted. Estimates were weighted using PATH Study Wave 4 longitudinal sampling weights for the Wave 1 cohort. Confidence intervals computed using the BRR method.

STable 8B. Dual use in 2022 among adults who smoked cigarettes in post-salt nicotine period (N=5,800).

Cigarette smoking status				95% CI	
2019	2022	n	%	lower bound	upper bound
Cig only	Cig only	2733	48.96	47.66	50.27
Cig only	Dual	477	7.92	7.06	8.87
Cig only	Neither	719	13.87	12.52	15.35
Cig only	Ecig only	210	3.56	3.02	4.18
Cig+nonfreq	Cig only	423	6.88	6.12	7.73
Cig+nonfreq	Dual	310	4.47	3.91	5.11
Cig+nonfreq	Neither	237	3.81	3.27	4.44
Cig+nonfreq	Ecig only	161	2.18	1.81	2.62
Cig+freq	Cig only	115	1.94	1.55	2.43
Cig+freq	Dual	181	2.86	2.40	3.42
Cig+freq	Neither	60	0.84	0.57	1.23
Cig+freq	Ecig only	174	2.70	2.31	3.15

Note. PATH Study Restricted Use Files used. Ns unweighted. Estimates were weighted using PATH Study Wave 7 longitudinal sampling weights for the Wave 4 cohort. Confidence intervals computed using the BRR method.
